# An evidence-based decision assistance model for predicting training outcome in juvenile guide dogs

**DOI:** 10.1371/journal.pone.0174261

**Published:** 2017-06-14

**Authors:** Naomi D. Harvey, Peter J. Craigon, Simon A. Blythe, Gary C. W. England, Lucy Asher

**Affiliations:** 1 School of Veterinary Medicine and Science, The University of Nottingham, Sutton Bonington Campus, Leicester, United Kingdom; 2 The Guide Dogs for the Blind Association, Hillfields, Burghfield Common, Reading, Berkshire, United Kingdom; 3 Centre for Behaviour and Evolution, Henry Wellcome Building, Newcastle University, Newcastle, United Kingdom; University of Missouri Columbia, UNITED STATES

## Abstract

Working dog organisations, such as Guide Dogs, need to regularly assess the behaviour of the dogs they train. In this study we developed a questionnaire-style behaviour assessment completed by training supervisors of juvenile guide dogs aged 5, 8 and 12 months old (n = 1,401), and evaluated aspects of its reliability and validity. Specifically, internal reliability, temporal consistency, construct validity, predictive criterion validity (comparing against later training outcome) and concurrent criterion validity (comparing against a standardised behaviour test) were evaluated. Thirty-nine questions were sourced either from previously published literature or created to meet requirements identified via Guide Dogs staff surveys and staff feedback. Internal reliability analyses revealed seven reliable and interpretable trait scales named according to the questions within them as: Adaptability; Body Sensitivity; Distractibility; Excitability; General Anxiety; Trainability and Stair Anxiety. Intra-individual temporal consistency of the scale scores between 5–8, 8–12 and 5–12 months was high. All scales excepting Body Sensitivity showed some degree of concurrent criterion validity. Predictive criterion validity was supported for all seven scales, since associations were found with training outcome, at at-least one age. Thresholds of z-scores on the scales were identified that were able to distinguish later training outcome by identifying 8.4% of all dogs withdrawn for behaviour and 8.5% of all qualified dogs, with 84% and 85% specificity. The questionnaire assessment was reliable and could detect traits that are consistent within individuals over time, despite juvenile dogs undergoing development during the study period. By applying thresholds to scores produced from the questionnaire this assessment could prove to be a highly valuable decision-making tool for Guide Dogs. This is the first questionnaire-style assessment of juvenile dogs that has shown value in predicting the training outcome of individual working dogs.

## Introduction

There is currently a large body of evidence supporting the existence of consistent individual differences in behaviour in many species including dogs [1,2]. Such differences are often referred to as personality, which depends upon animals exhibiting behavioural differences that are consistent within the individual as compared to the rest of the population. Accurate assessment of such individual differences in dog behaviour could be of great value to working dog organisations where individuals with certain attributes are required (e.g. [3] in addition to rescue shelters that want to increase the adoptability of dogs in their care (e.g. [4].

Guide Dogs, UK (formerly Guide Dogs for the Blind Association, UK) is the largest dog breeding and training school in the world [5], breeding approximately 1,400 dogs per year. Within Guide Dogs, puppies are placed with volunteer carers from the age of 2 to 14 months of age (a stage known as puppy walking), after which they enter formal training. Ideally, individuals not suitable to the guiding role would be identified before they enter formal training, which is the mostly costly and resource-consuming phase of training. For this reason, the behaviour of each dog is routinely assessed at the age of 6–8 weeks using a standardised puppy behaviour test [5]. Using this test Guide Dogs aim to remove 3% of dogs from the program at this stage, whilst all others enter puppy walking (Whiteside, H., 2014. personal communication). In 2013, 27% of dogs that entered puppy walking (n = 352) were withdrawn from the training program for behavioural reasons, with 72% of these being withdrawn during the formal training stage, by which point they have already incurred large financial and time investments.

Predicting adult behaviour from juvenile animals is challenging due to the impact of continuing neurological, environmental and genetic interactions on behavioural development [6,7]. However, previous research indicates that behaviour of developing animals, including dogs, becomes more predictive and consistent with age [8,9]. It is therefore possible that assessment of dog behaviour conducted at later stages of development than conventional puppy tests, which typically occur between 6–12 weeks of age, could provide a more valid and predictive profile of the individual [10]. Behaviour assessments of dogs in the juvenile period of development (occurring between approximately 3 to 12 or 24 months, depending on breed; [11]) have shown the potential to be able to predict working dog outcomes [10,12,13]. Accurate assessment of a dog’s behaviour during the juvenile stage of development could be valuable in more ways than just predicting working dog suitability. An accurate and reliable behavioural profile of an individual dog, evaluated at regular time intervals, could allow for targeted training interventions for dogs predicted as likely to be withdrawn, enable better matching between a dog and their future guide dog owner or re-homer, as well as assisting in monitoring population level behavioural trends in response to changes in training or breeding practices.

A standardised behaviour test of 5 and 8 month old juvenile guide dogs [10] has shown promise in predicting future outcomes; however, such a test may not be cost or time effective for use as a regular behaviour assessment tool. Standardised behaviour tests also fail to capture infrequent behaviour and behaviour occurring outside the test environment. For these reasons, a questionnaire-based assessment completed by the dog’s trainer or supervisor at regular intervals may be preferable. A questionnaire-based assessment (known as the C-BARQ) completed by a dog’s puppy raiser (the US equivalent of a puppy walker) at 6 and 12 months of age has been shown to be predictive of guide dog training success in US guiding agencies [13]. Puppy walking may represent the most valid time period to assess and predict the future behaviour of a working dog; it is a time of relative stability when the dogs inhabit a human home and experience routines most similar to those they will encounter when they are qualified and working.

The main aim of this study was to develop and validate a questionnaire-based assessment of dog behaviour and personality in juvenile guide dogs that could be completed by their training supervisors. The questionnaire was designed to address: (1) the main behavioural reasons for withdrawal within Guide Dogs; (2) behaviour of importance to guide dog owners; (3) behaviour important to success in the guiding role. It is important to demonstrate evidence of reliability and validity for any new behaviour assessment [14]. Many published dog behaviour assessments only present the predictive criterion validity of their tests (e.g. [5,12,13,15–17]). However, without also evaluating the concurrent criterion validity of a test by comparing against another independent measure taken at the same time as the assessment in question, it is difficult to decipher whether a lack of predictive validity is due to the assessment not being valid or to the behaviour in the animal actually changing over time. Concurrent criterion validity may be of particular importance when validating an assessment of a juvenile animal due to the dynamic nature of behavioural development. For this reason a further aim of this study was to evaluate the level of convergence and divergence between the scores on the designed assessment and scores given to the same dogs on the juvenile guide dog behaviour test described in [10]. Due to the large number of differences in the methods of measurement, even low levels of convergence between the two methods would be considered evidence of concurrent criterion validity [18–21]. Finally, this new questionnaire would be subject to the operational constraints of the end user (Guide Dogs, UK) and needs to be designed for them to use in a business-as-usual manner to aid in their understanding of the behavioural phenotypes of the dogs in their system. For this reasons, a decision-assistance tool was designed that would alert the end user to dogs scoring within ‘at risk’ or ‘safe’ zones for each behavioural trait. This system was designed and tested on data from the behaviour questionnaire collected on one year’s worth of trainee guide dogs in order to represent the full range of genetic and behavioural variance within the organisation, and recommendations for its use are made.

## Methods

This study was conducted according to the University of Nottingham's institutional guidelines and received ethical approval from the School of Veterinary Medicine and Science’s ethics committee, who acted as the Institutional Review Board. Participants were members of Guide Dogs training staff and Guide Dogs managers disseminated the assessment internally, with dog behavioural data collected as part of business as usual practice. During questionnaire development, training staff were consulted for feedback on their needs for a new system and were recruited via email, with no obligation to participate, their information was anonymised and they were able to withdraw at any time. Informed consent was received verbally or given when completing an online feedback form. All data was analysed by the Nottingham research team only, given to us in anonymised format, and results were not shared with Guide Dogs until after all dogs had completed training; as such the results did not inform any decisions made about the dogs progression in training.

### Subjects

**Fig 1:**
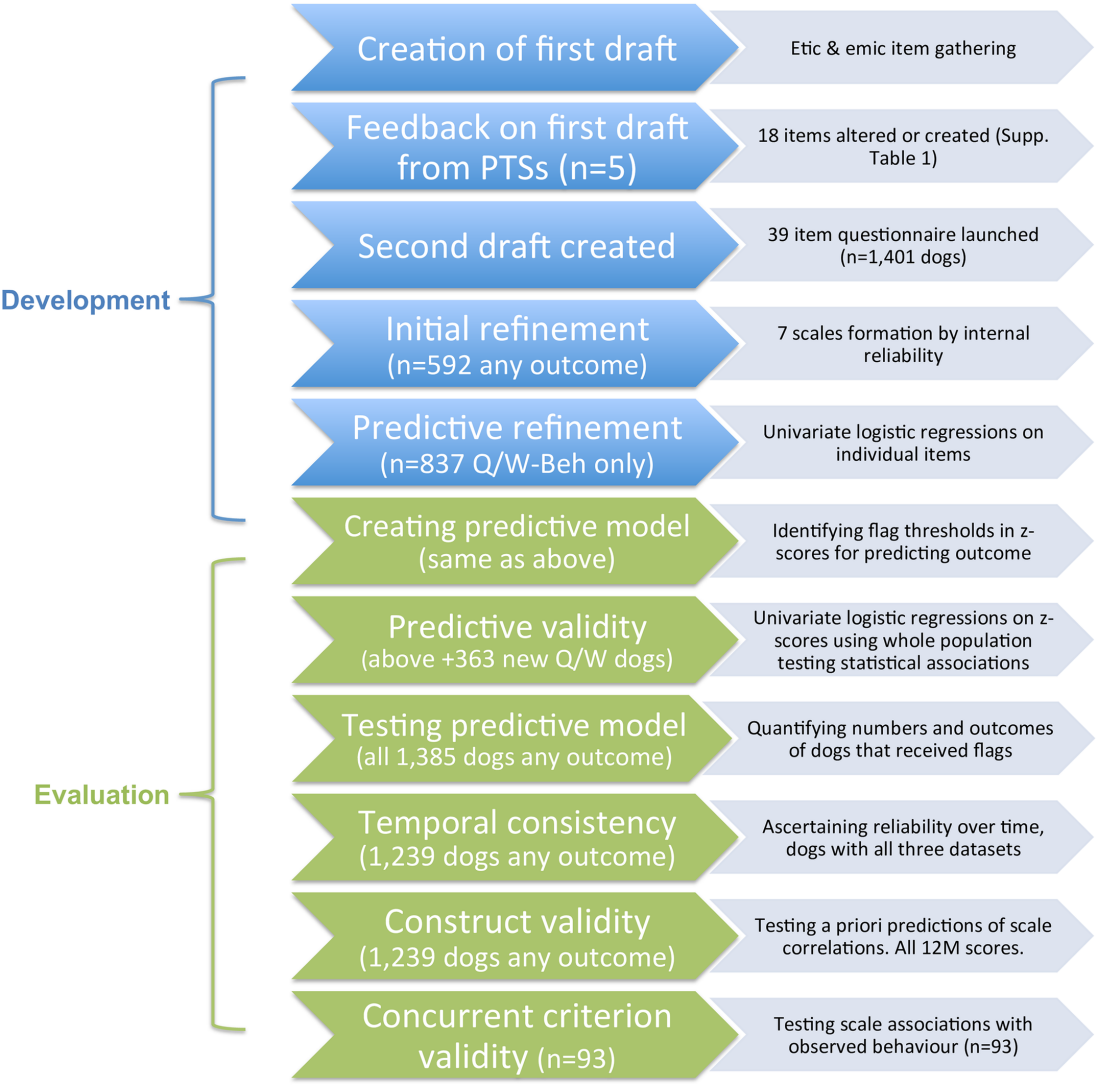
Flow chart depicting the various stages of questionnaire development and evaluation, each given with their respective aims and sample sizes.

All dogs in the Guide Dogs programme born between 6/12/2011–1/1/2013 were included in this study (n = 1,401) coming from 201 litters, with 64 sires and 195 dams, with 1,359 bred by Guide Dogs. A total of 54 puppy training supervisors supervised the dogs in this study. The breed profile of the sample included a mixture of pure and crossbreeds (sire x dam): Golden retriever x Labrador (n = 469); Labrador (n = 401), Labrador x Golden retriever crossbreed (n = 131), Golden retriever (n = 128), Labrador x Golden retriever (n = 63), German shepherd dog (n = 51), Golden retriever x German shepherd dog (n = 88), Labrador x Labrador crossbreed (n = 25), Golden retriever x Golden retriever crossbreed (15), Golden retriever x Flat coated retriever (n = 14). Puppy training supervisors visited the dogs every 1–2 months to monitor their progress and were required to complete the questionnaire within 1 week of the dogs turning 5, 8 and 12 months of age. Different sections of this study utilised different subsets of this overall sample for statistical analysis (see Table 1 and Fig 1 for a breakdown of samples involved in each analysis). The 1,401 dogs in this sample left puppy walking and entered training when they were a mean of 14.4 months of age (SD ± 1.2), whilst those withdrawn for behaviour were withdrawn at a mean age of 17.0 months (SD ± 3.9), and those that qualified did so at a mean age of 22.0 months (SD ± 1.7). Dogs selected for breeding did not enter training, and whilst they could be considered to be successful, due to the complexity of breeding selection and the differing experiences of these dogs they were not included as ‘qualified’ for the purposes of this study. Of the 1,401 dogs included the study, 816 went on to qualify as guide dogs, 384 were withdrawn for behaviour, 103 were withdrawn for health, 74 were selected as breeding stock, whilst 8 were withdrawn for both health & behaviour, 8 died during the study period and 8 were transferred to external organisations.

**Table 1:** Breakdown of samples included in each step of development and evaluation.

Stage	N (M:F)	N	Dogs included in sample	Dogs excluded
**Development**				
Initial refinement: scale formation by internal reliability	592 (306:286)	5M = 592 8M = 584 12M = 553	All dogs in the Guide Dogs programme that turned five months of age between May 1^st^ and November 3^rd^ 2012	None
Predictive refinement	837 (449:388)	5M = 837 8M = 832 12M = 811	Dogs born between 6/12/2011 and 1/9/2012 that later qualified (580) or were withdrawn for behaviour (257)	Withdrawn for health (72), withdrawn for health & behaviour (6), selected for breeding (52), transferred to external organisations (8) or deceased (8)
**Evaluation**				
Creating predictive model	837 (449:388)	5M = 837 8M = 832 12M = 811	Dogs born between 6/12/2011 and 1/9/2012 that later qualified (580) or were withdrawn for behaviour (257)	Withdrawn for health (72), withdrawn for health & behaviour (6), selected for breeding (52), transferred to external organisations (8) or deceased (8)
*Predictive criterion validity*:				
Testing thresholds, PPV and sensitivity	1,385 (717:668)	5M = 1,385 8M = 1,275 12M = 1,239	All dogs born between 6/12/2011–1/1/2013 that later qualified as a guide dog (816), were withdrawn for behaviour (384), withdrawn for health (103), withdrawn for health & behaviour (8) or selected as breeding stock (74)	Transferred to external organisations (8) or deceased (8)
Testing statistical associations	1,200 (645:555)	5M n = 1,200 8M n = 1,131 12M n = 1,103	All dogs born between 6/12/2011–1/1/2013 which qualified as a guide dog (816) or were withdrawn for behaviour (384)	Withdrawn for health (103), withdrawn for health & behaviour (8), selected for breeding (74), transferred to external organisations (8) or deceased (8)
Temporal consistency and Construct validity	1,239 (643:596)	NA	Data from dogs with questionnaires completed at 12M (for temporal consistency data for these dogs from 5M and 8M was also utilised)	Transferred to external organisations (8) or deceased (8)
Concurrent criterion validity	93 (41:52)	5M = 82 8M = 80	Dogs which had complete questionnaire and practical behaviour test data	None

### Questionnaire development

A questionnaire utilising behavioural descriptive items was developed for completion by Guide Dogs training staff using the stages outlined in Fig 1. Development of the questionnaire incorporated information gained from: 1) reviewing relevant published literature, 2) a national survey of Guide Dogs training staff and 3) content analysis of ‘free text’ sections of guide dogs behaviour reports to identify the behaviours recorded most often and the language used.

The questionnaire was developed using both emic (creation of new scales based on in-depth knowledge of target species/culture/population) and etic (direct application of existing assessments/items designed for a different species/culture/population) approaches [1,21]. Where possible relevant question items (items) were sourced from previous questionnaires that had demonstrated predictive validity for guide dogs [13,15,22,23] and a questionnaire study undertaken by Guide Dogs in 2006/2007. Additional items were then designed by the authors to cover all areas of importance specific to Guide Dogs. A draft questionnaire was refined based on feedback from a panel of Guide Dogs’ puppy training supervisors (PTSs, n = 5 from different regions of the UK) on the questionnaires applicability to the behaviour of juvenile guide dogs and the needs of the organisation; thereby addressing the questionnaires content validity [24].

The questionnaire, referred to as the puppy training supervisor questionnaire (PTSQ), contained a total of 39 items; 36 items expected to form nine targeted ‘traits’, and 3 miscellaneous items (S1 Table). Most items were behavioural descriptions, but a small number of adjective items were also included. Items were scored using a 100mm long visual analogue scale (VAS), using the anchors ‘Never’ and ‘Almost Always’ for behavioural descriptive items, and ‘Really does not describe this dog’ to ‘Really describes this dog’ for adjective items. The number of millimetres between the left anchor and the mark made by the rater was the response value. A non-sectioned, randomised approach was chosen for the arrangement of the items to reduce potential order effects.

### Questionnaire evaluation

The questionnaire was distributed using an online system created by Guide Dogs. Automatic requests and reminders were generated when the dogs were 21, 34, and 51 weeks of age (1 week prior to turning 5, 8 and 12 months of age). Henceforth, assessments completed at these age brackets will be referred to as 5M, 8M, or 12M questionnaires. The PTSs were instructed to complete the questionnaire using their knowledge of the dogs’ behaviour over the previous three months. Each question required an answer before the PTS was able to move onto the next page. Where the PTS had insufficient information to answer an item they were requested to speak with the dog’s puppy walker (volunteer carer who lives with the dog) to gain relevant information or were able to select a ‘Not Known’ option, and were requested to state why the information was not known in a free-text box.

A sub-sample of dogs also completed a practical behaviour test at 5 and 8-months of age, which was subsequently scored using a behavioural coding approach. Full details can be found in [10] but in brief the sample consisted of 93 individual dog-PW dyads with 69 tested twice (13 attended only the first test, and 11 only the second test). The test consisted of 11 subtests covering responses to: meeting a stranger; basic obedience with a familiar handler (the dogs PW) and then with a stranger (STR; the experimenter), body sensitivity checks (handling/petting, gentle placement of a tea-towel on their back and inserting their head into a ring in return for a treat); and four types of distractions including food on the ground, a moving toy ‘robin’, stationary pigeon decoys and an unfamiliar human. Definitions of coded behaviour from the subtests included in this analysis can be found in S1 Table. Unfortunately the test could not be repeated when dogs were 12 months of age due to logistical and resource limitations.

### Data analysis

With regards to terminology, the term ‘component’ is used to indicate a grouping of items identified by PCA; the term ‘component’ will be used when discussing assessment of the PCA results only. The term ‘scale’ will be used to indicate a grouping of items that have subsequently been shown to significantly inter-correlate and meet standards for internal reliability at all ages assessed. Unless stated otherwise, all analysis was conducted in SPSS v. 21 (SPSS Inc., Chicago, IL, USA).

#### Development—Initial refinement

All question items were designed to assess a specific aspect of dog personality or behaviour. Seven targeted ‘traits’ were considered to be covered by two or more items in the PTSQ: Attentiveness; Body Sensitivity; Distractibility; Energy/Immaturity; Excitability; General Anxiety; and Trainability, along with 5 items that were considered to be miscellaneous (see S2 Table). Principal components analyses (PCA) were first performed for the questionnaires at each of the three ages to test the expected structures (n = 592). Each age was assessed in order to account for and evaluate the stability of the scale structures with dogs of different ages, who were undergoing development, which may cause their behaviour to be inconsistent in its expression at different ages. The PCA’s were based upon eigenvalues >1, with varimax rotation, using a correlation matrix with loadings above 0.40 considered most salient [25] Items that loaded on more than one component were removed from the component upon which it showed the weakest loading.

Internal reliability for stable component structures (those consistent in the items loading within them at two or all three ages) was estimated via Cronbach’s alpha. For items that loaded inconsistently (i.e. the items within them loaded on different components at different ages) across the PCA’s, an exploratory Cronbach’s alpha analysis was conducted to identify reliable groupings using the data from the 12M PTSQ, and identified groupings were later tested for reliability on the 5M and 8M data. The 12M PTSQ was chosen as behaviour is considered to be more stable with age [8,9]. There were three steps to this method: (1) a reliability coefficient (alpha) was calculated for a single large group comprising all inconsistently loading items; (2) items were identified, which if removed would increase the groups reliability, and items were sequentially removed if their removal increased the alpha by >0.05; (3) item removal ceased when further item removal no longer improved the reliability coefficients, and remaining items were considered to be a new grouping of significantly correlated items. The process was repeated for all remaining items that loaded inconsistently in the PCA’s until no further groupings could be formed. All groupings identified in this way were then tested for internal reliability in the 5M and 8M data sets, and accepted as new scales only if they achieved alpha values of >0.70 in every dataset. Scales were named based upon the ‘traits’ that the items within them were originally designed to assess.

#### Development—Predictive refinement

Each item from within the scales, at each age, was initially tested for statistical association with later training outcome. Univariate binary logistic regressions were used to compare each item to the dependent variable: qualified as a guide dog or withdrawn for behavioural reasons. Items that did not achieve predictive significance (p<0.05) at any age were removed from the scale to which they had belonged.

#### Evaluation—Creating a predictive model

Mean scores were created for all finalised scale structures; where an item showed a negative correlation to the other items on the scale it was transformed using: ‘100 minus raw item score’, and the transformed item score was used to calculate the mean scale score.

Normalised z-scores were created and used in all predictive analyses. Z scores were made using the ‘ddply’ and ‘scale’ commands in R version 3.0.2 (R Core Team, 2013) according to the following calculation:

Z=(raw score−population mean)/population standard deviation

Three types of z-scores were made using means and standard deviations from: 1) the whole sample, for an uncontrolled score, 2) within breed, to control for breed, 3) within breed and sex to control for breed and sex. Binary logistic regression analyses were performed for the purpose of ascertaining statistical association (n = 837). One regression analysis was conducted for the 5M, 8M and 12M assessments with each type of z-scores for each scale as the dependent variable and training outcome as the independent variable.

**Fig 2:**
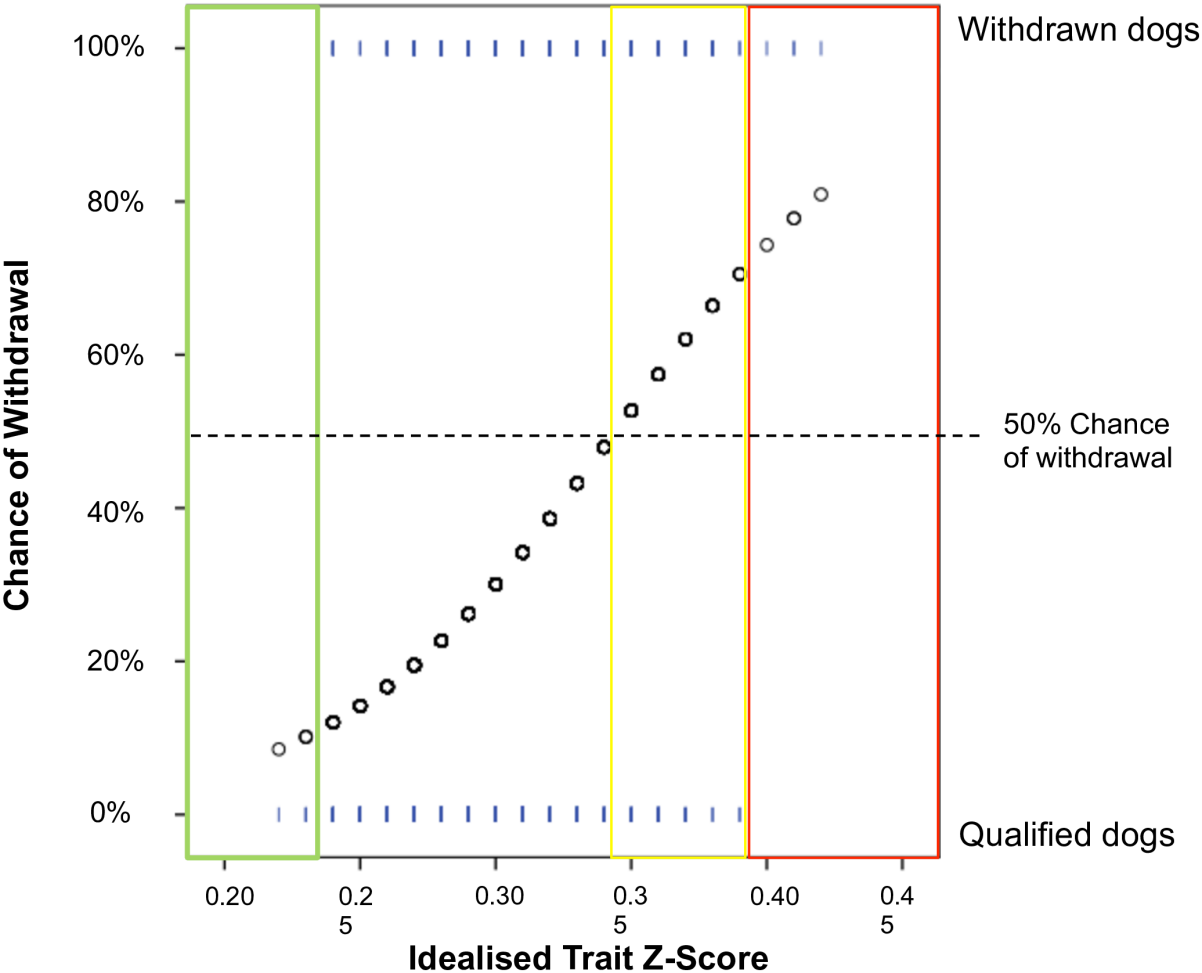
Theoretical probability plot showing an idealised association between a trait score and later training outcome. The green box surrounds a range of the score within which only qualified dogs scored, the yellow box indicates dogs who’s scores would be within the range for a greater than 50% chance of withdrawal but not extreme, whilst the red box indicates the range of the score where only dogs that were later withdrawn scored, representing those extremely unsuitable to guiding. Cut-offs were identified in the scores that marked the edges of these zones, and green, yellow and red flags assigned accordingly to dogs that fell within them.

In order to create a system that would alert the end user to individual ‘at risk’ dogs a series of cut-offs was identified to which ‘flags’ could be assigned. Visual assessment of probability plots was used to ascertain values of z-scores and probabilities above or below which only dogs that later qualified or were withdrawn scored; thus identifying 100% correct cut-off points for prediction. The z-score type (whole population, within breed, or breed and sex) that resulted in the best separation of individual dogs between outcomes was used for creating a flag system to alert dogs at highest and lowest risk of later withdrawal. Scales where thresholds could be found which identified qualified or withdrawn dogs with no false positives were considered to demonstrate real-world predictive ability. Thresholds at which dogs received a >50% probability of withdrawal were also identified. A decision assistance model was created based on these thresholds. This model contained three basic rules: 1) all dogs scoring within the threshold for identifying qualified dogs were assigned ‘green’ flags; 2) all dogs scoring within the threshold for identifying withdrawn dogs were flagged ‘red’; and 3) all dogs which achieved a probability of being withdrawn of more than 50%, but scored below the threshold for red flags, were assigned ‘yellow’ flags. A dog could receive no flag for a trait, in which case its score on that trait cannot predict its outcome, or a flag for more than one trait at more than one age. An idealised example of how the flag system could work given a perfect distribution of a single trait score can be seen in Fig 2.

#### Evaluation—Predictive criterion validity

Binary logistic regression analyses were performed to ascertain the statistical association of the scales with later training outcome using data on all dogs that had an outcome of qualification or withdrawn for behaviour. Seven univariate logistic regression analyses were performed for each age, one for each scale, where qualified or withdrawn for behaviour was the outcome (DV) variable with breed and sex controlled for by inclusion as fixed effects. Multivariate regressions were also attempted, using a forwards stepwise approach for all scores which exhibited significance to p<0.05, in order to investigate whether the scores could work in an additive manner.

In order to test how the flag-system could work for all dogs, a new set of z-scores was created based on population statistics for all dogs, including those with later outcomes different to qualification or withdrawal for behaviour, plus an additional 1/3 of dogs not previously utilised (total n = 1,385, including 548 new dogs). Following creation of new z-scores from the new population means and standard deviations, those individuals whose scores fell within or outside of the previously identified thresholds were ‘flagged’ accordingly. This process was repeated for the 5, 8 and then 12-month datasets and the number, and eventual outcome, of flagged dogs within each dataset were summed. The positive predictive value (PPV) and sensitivity of this dataset was then evaluated [14]. PPVs were the number of correctly predicted outcomes divided by the total number of dogs with that outcome. Sensitivity is the number of correct predictions of an outcome, divided by the total number of predictions made for that outcome.

#### Evaluation—Temporal consistency

Spearman’s Rank correlation coefficients were used to investigate bivariate rank order consistency between scores given to the same dogs at all three ages [26,27]. Given the intrinsic link between p-values and sample size [28], and the large sample involved here, effect size was considered instead of statistical significance. Correlations above 0.30 were considered acceptable, as the mean level of temporal consistency found in dogs less than 12 months of age is 0.34 [26].

#### Evaluation—Construct validity

Only the 12M questionnaire scores were used here to avoid pseudo-replication. The majority of the scales showed a non-normal distribution, therefore Spearman’s Rank correlations were utilised to create a correlation matrix. Predicted correlations between the finalised scales were formed *a priori* based upon the traits they were considered to assess. These predicted associations, both convergent (positive) and divergent (negative), could be considered as individual hypotheses regarding which scales should theoretically be associated with one another [24]. Effect size was considered instead of statistical significance, with above medium sized correlations of >0.4 used as criteria for acceptance of a hypothesis.

#### Evaluation—Concurrent criterion validity

In order to try and reduce the number of variables tested, mean obedience response scores were made for the dog’s response to the ‘Sit’, ‘Wait’ and ‘Down’ commands within subtests 2 and 3 (PW and stranger obedience) creating a score where higher numbers indicate slower responses, and a single score was made from the variable measured in subtest 6 –Head Ring. The ‘Head ring score’ was formed by summing the dogs results from the two repetitions, with numerical representatives of each response assigned so that lowest numbers represented least adverse reactions (tail height: Up = 0, Half Up = 1, Neutral = 2, Low = 3; ear position: Neutral = 0, backwards = 1; body posture: neutral = 0, stretched = 1) creating a score for each dog with a possible range of 0–10. Three separate principle components analysis (PCA) were then applied to groups of variables within 1) subtest 7 –Tea Towel, 2) subtests 8–11 –Distractions and 3) for behavioural events counted throughout the entire test. PCAs were based on Eigen values >1, using varimax rotation and values of >0.50 were considered salient [25].

Predictions were made *a priori* about which behavioural coding measures would be associated with which questionnaire scale scores, as is important for evaluation of criterion validity [24]. Each prediction used a scale score from the questionnaire (a 0-100mm continuous measure) as the dependent variable, with behavioural coding measures from the juvenile guide dog behaviour test [10] as independent variables (IV’s). The predictions were based on the shared areas of behaviour the test and questionnaire were designed to assess. A total of 42 predicted associations were made between the behavioural coding measures and six of the questionnaire scales (see S3 Table). These were tested using non-parametric statistics for all comparisons: Mann-Whitney U-tests were performed with all binary variables; Kruskal Wallis tests were conducted for categorical data; and Spearman’s Rank correlations coefficients were calculated for continuous IV’s.

## Results

Due to dogs being withdrawn, transferred to other roles within or outside Guide Dogs, the exact numbers of dogs reduced at 8M and 12M. Different subsamples of dogs were used for different analyses; numbers involved in each analysis will be given alongside relevant data and can be seen in Table 1. At the time of PTSQ completion the age of dogs in the sample was: 5.13 months (mean 156 days ± 7 days SD); 8.10 months (mean 245 days ± 6 days SD); and 12.01 months (mean 364 days ± 6 days SD).

### Development—Initial refinement

Seven scales (groups of reliably correlated items) could be formed from the questionnaire; these were named according to the items within them, and the dog personality dimensions they were designed to assess, as: Adaptability; Body Sensitivity; Distractibility; Excitability; General Anxiety; Trainability and Stair Anxiety (Table 2). Seven of the scales identified were formed from the PCA analyses (see S4 Table for PCA loadings), whilst two (Trainability and Excitability) were formed from exploratory Cronbach’s alpha analyses.

**Table 2:** The final PTSQ scales and miscellaneous items formed based upon PCA and internal reliability analysis of dogs at three different ages: 5M (n = 592); 8M (n = 584); 12M (n = 553).

Scale	Item Wording	Direction	Designed to assess
**Trainability**	Attention can be attracted easily but it loses interest soon	-	Attentiveness
	Attention can be easily distracted	-	Attentiveness
	Is stubborn	-	Misc.
	Seems not to listen even if it knows someone is speaking to it	-	Trainability
	Refuses to obey commands, which in the past it has proven it has learned	-	Trainability
	Needs obedience commands repeating to get a response	-	Trainability
	Is attentive to you	+	Attentiveness
	Shows a rapid response to correction by handling	+	Trainability
	Is easy to control	+	Trainability
	Is eager to please	+	Trainability
	Is friendly	+	Misc.
	Stays/waits when instructed to	+	Trainability
	Responds immediately to the recall command when off lead	+	Trainability
**General Anxiety**	Is obviously startled by loud or unexpected sounds	+	Anxiety
	Is obviously startled by odd or unexpected things or objects	+	Anxiety
	Is anxious or uneasy in new situations	+	Anxiety
	Backs away from or is reluctant to pass objects on the street (such as collecting boxes, bin bags or children's ride-on toys)	+	Anxiety
**Adaptability**	Adapts well to new situations and environments	+	Adaptability
	Recovers quickly after being unsettled or frightened	+	Adaptability
**Excitability**	Exhibits a high degree of excitement (jumps up; barks; coughs etc.) when goes somewhere new	+	Excitability
	Exhibits a high degree of excitement (jumps up; barks; coughs etc.) when you initially enter the home	+	Excitability
	Is active and energetic	+	Immaturity
	Is mischievous	+	Immaturity
	Is calm and quiet	-	Excitability
	Is initially excitable (jumps up; barks; coughs etc.), but quickly settles	+	Excitability
**Body Sensitivity**	Is uneasy with being physically handled/groomed	+	Body Sensitivity
	Appears uneasy or uncomfortable when putting on Guide Dog equipment (including collars)	+	Body Sensitivity
	Is reluctant to walk close to the handler	+	Body Sensitivity
**Distractibility**	Pulls (including lunging) towards unfamiliar dogs	+	Distractibility
	Pulls towards/distracted by food on the ground or food scents	+	Distractibility
	Shows interest (attempts to greet, sniffs, wags tail) when passing children or members of the public	+	Distractibility
	Shows interest (attempts to greet, sniffs, wags tail) when it encounters other dogs	+	Distractibility
	Attempts to sniff objects in the street	+	Distractibility
**Stair Anxiety**	Appears uneasy on closed stairs	+	Anxiety
	Appears uneasy on open or unusual (e.g. glass) stairs	+	Anxiety
**Miscellaneous**	Requires an indoor kennel when left alone	NA	Misc.
	Readily accepts the responsibility of decision making (12M only)	NA	Trainability
	Will look at you when you talk to it directly in the home environment	NA	Attentiveness
	Shows interest (attempts to greet, sniffs, wags tail) when directly approached by children or member of the public	NA	Distractibility

Overall, the scales achieved good (>0.70) to high (>0.80) levels of internal reliability, at all ages; the only exception being Body Sensitivity, which achieved a mean alpha value of 0.58 (Table 3).

**Table 3:** Reliability statistics for final PTSQ scales. Cronbach’s alpha statistics are provided for internal reliability at each age and a mean across the ages. Scales that were formed using Cronbach’s alpha analyses of the 12-month data, from items that loaded inconsistently in the PCA’s, are indicated in the Scale column by an asterisk (*). Spearman’s correlations between ages are provided for temporal consistency (*** p< 0.001).

	Internal reliability				Temporal consistency (n = 1,239)		
Scale	5M (n = 1,401)	8M (n = 1,288)	12M (n = 1,239)	Mean	5-8M	8-12M	5-12M
Trainability*	0.82	0.87	0.88	0.86	0.63***	0.69***	0.55***
Distractibility	0.85	0.84	0.84	0.84	0.57***	0.56***	0.43***
General Anxiety	0.84	0.85	0.83	0.84	0.53***	0.59***	0.42***
Excitability*	0.79	0.80	0.80	0.80	0.62***	0.68***	0.51***
Stair Anxiety	0.79	0.83	0.84	0.82	0.56***	0.63***	0.44***
Adaptability	0.76	0.74	0.79	0.76	0.60***	0.63***	0.51***
Body Sensitivity	0.55	0.56	0.62	0.58	0.57***	0.65***	0.50***

### Development—Predictive refinement

All but two items were significantly associated (p<0.05) with withdrawal for at least one age (see S5 Table). The removal of the two items from the scales to which they contributed, one from Trainability (‘Will look at you when you talk to it directly in the home environment’) and one from Distractibility (‘Shows interest (attempts to greet, sniffs, wags tail) when directly approached by children or member of the public’) did not change the scales’ mean scores or internal reliability so were removed from further analyses.

#### Evaluation—Creating predictive model

No scores could be combined into a multivariate model, indicating that the scores did not function to predict withdrawal in an additive manner. For this reason all scores, at each age were used as separate predictors of training outcome.

With regards to predicting the later outcome of individual dogs from 5 months of age, five scores were able to distinguish individuals later withdrawn for behaviour (Trainability; General Anxiety; Excitability; Body Sensitivity and Distractibility) and five were able to distinguish individuals that later qualified (Trainability; General Anxiety; Excitability; Distractibility and Adaptability). Adaptability and Stair Anxiety were not able to distinguish dogs that went on to be withdrawn from scores given this age, and only Adaptability Z-scores controlling for breed and sex could identify individuals that went on to qualify.

When scored at 8 months of age, all scores except Distractibility were able to distinguish between dogs with different future outcomes. Trainability scores at this age showed no predictive ability with regards to identifying dogs that were withdrawn, but they were able to distinguish dogs that went on to later qualify. All other scales (General Anxiety, Adaptability, Excitability, Body Sensitivity and Stair Anxiety) were able to distinguish individuals that were withdrawn. The only trait at this age that did not highlight qualified individuals was Body Sensitivity.

All scores from dogs 12-months of age, except from Stair Anxiety and Adaptability, were able to identify individual withdrawn dogs, whilst four (Trainability, General Anxiety, Excitability and Distractibility) were able to separate individual qualified dogs. The best z-score for prediction (uncontrolled, controlled for breed, controlled for breed and sex) differed between scales and between flag type being assigned (see S6 Table). An example of how the flag system worked for identifying dogs that would go on to be withdrawn and those at high risk can be seen in S1 Fig using scores from General Anxiety at 8-months.

### Evaluation—Predictive criterion validity

Logistic regressions comparing the raw questionnaire trait scales to qualified or withdrawn dogs from the entire dataset confirmed the predictive validity of the scales (Table 4). Breed was significant in the majority of models at 5-months of age, but not at 8 or 12-months, whilst sex was not significant in any predictive model.

**Table 4:** Results for 21 logistic regression models comparing the questionnaire trait scores against training outcome for all dogs. Training outcome (qualified or withdrawn for behaviour) served as the dependent variable whilst scale score, sex and breed were included in each model as independent variables. Wald statistics provided as a measure of effect size. Sample utilised all dogs that qualified or were withdrawn for behaviour from the full cohort (5M n = 1,207[816Q:391WB], 8M n = 1,131[770Q:361WB], 12M n = 1,103[768Q:335WB]).

Age (months)	Trait	P	Wald	OR	95% CI
5	Trainability ^b^	<0.001	14.84	0.980	(0.970, 0.990)
5	Distractibility ^a^	0.328	0.96		
5	General Anxiety ^a^	0.001	10.63	1.015	(1.006, 1.025)
5	Adaptability ^b^	0.012	6.35	0.992	(0.986, 0.998)
5	Excitability ^a^	<0.001	13.43	1.013	(1.006, 1.020)
5	Stair Anxiety	0.010	6.58	1.009	(1.002, 1.016)
5	Body Sensitivity ^a^	<0.001	14.95	1.021	(1.010, 1.032)
8	Trainability ^b^	<0.001	17.24	0.98	(0.970, 0.989)
8	Distractibility ^b^	0.434	0.61		
8	General Anxiety	<0.001	22.59	1.023	(1.014, 1.033)
8	Adaptability	<0.001	34.89	0.098	(0.974, 0.987)
8	Excitability ^b^	<0.001	18.32	1.015	(1.088, 1.022)
8	Stair Anxiety	0.017	5.69	1.010	(1.002, 1.018)
8	Body Sensitivity ^b^	<0.001	14.3	1.022	(1.011, 1.034)
12	Trainability	<0.001	20.88	0.978	(0.968, 0.987)
12	Distractibility	0.011	6.54	1.008	(1.002, 1.014)
12	General Anxiety	<0.001	25.34	1.027	(1.016, 1.038)
12	Adaptability	<0.001	49.1	0.977	(0.971, 0.983)
12	Excitability	<0.001	18.65	1.016	(1.009, 1.023)
12	Stair Anxiety	0.043	4.09	1.010	(1.000, 1.019)
12	Body Sensitivity	<0.001	18.81	1.024	(1.013, 1.036)

Note:

^a^ Breed was significant to P<0.05,

^b^ Breed was approaching significance at P <0.1

#### Testing flag thresholds

**Fig 3:**
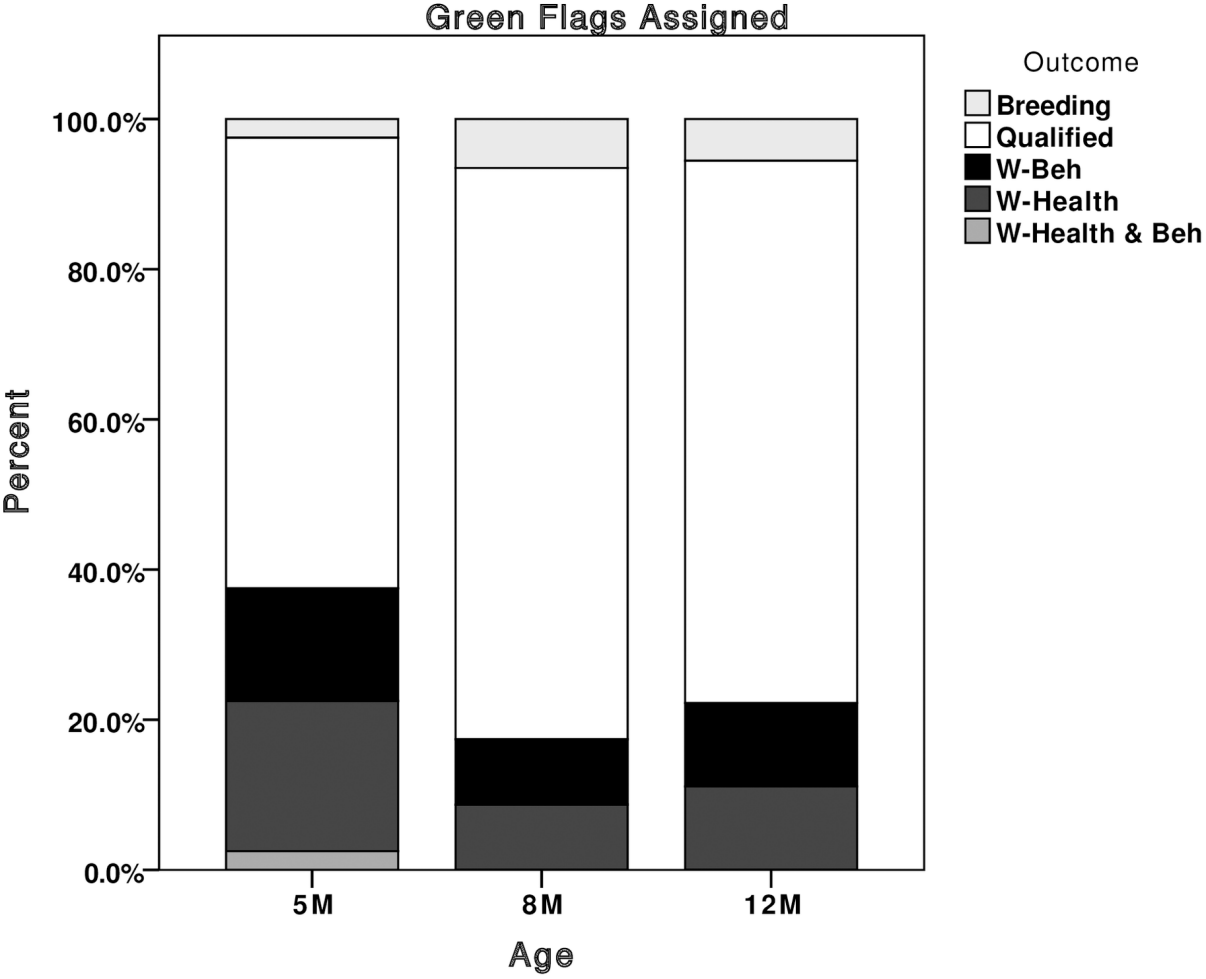
Stacked bar chart showing the percentage of green flags that would have been given out at each assessment, broken down by the dog’s final training outcome. Breeding, dogs selected as breeding stock; Qualified, dogs that qualified as working guide dogs; W-Beh, dogs withdrawn for behavioural reasons; W-Health, dogs withdrawn for health reasons; W-Health & Beh, dogs marked as withdrawn for both health and behavioural reasons.

**Fig 4:**
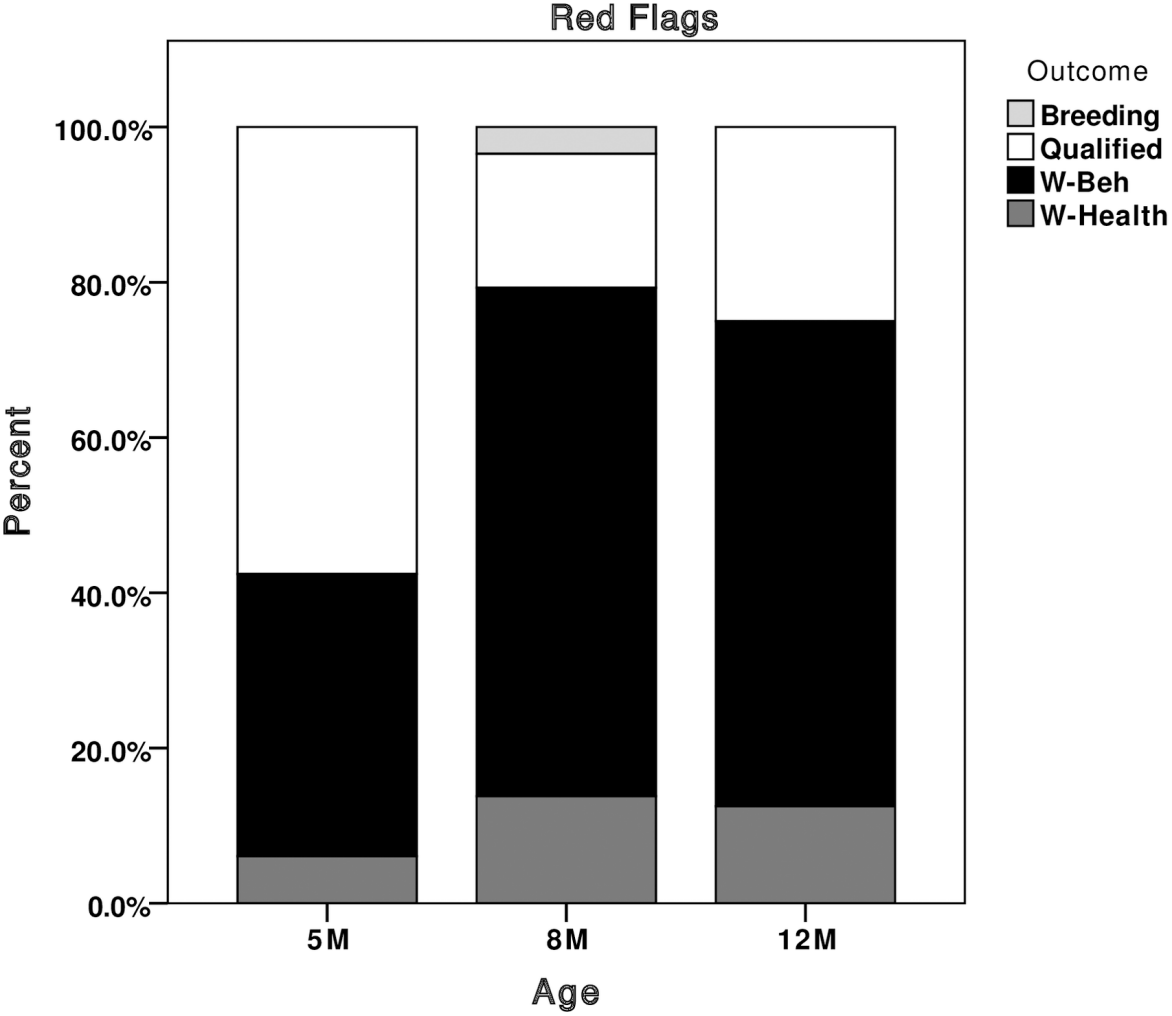
Stacked bar chart showing the percentage of red flags given that would have been out at each assessment, broken down by the dog’s final training outcome. Breeding, dogs selected as breeding stock; Qualified, dogs that qualified as working guide dogs; W-Beh, dogs withdrawn for behavioural reasons; W-Health, dogs withdrawn for health reasons.

**Fig 5:**
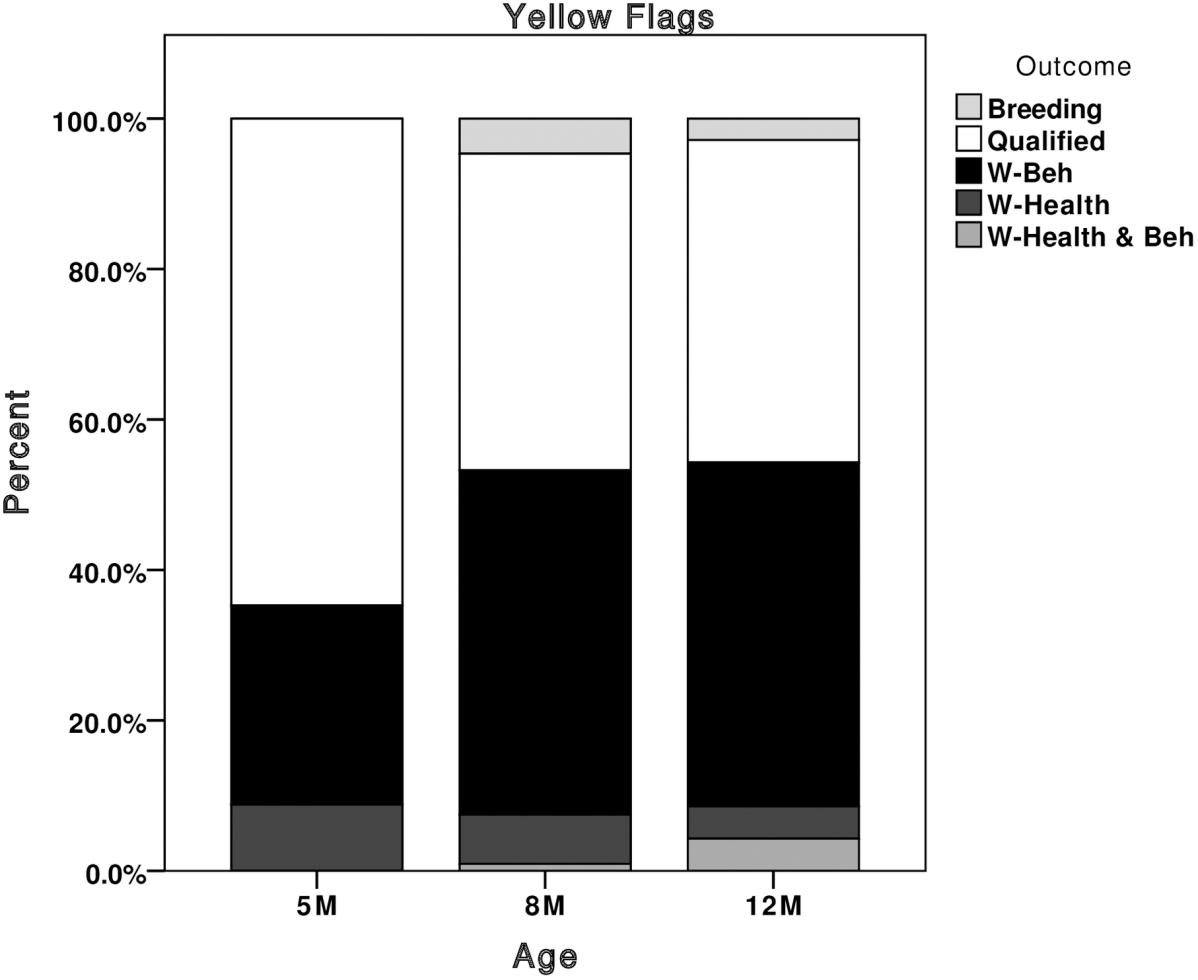
Stacked bar chart showing the percentage of yellow flags that would have been given out at each assessment, broken down by the dog’s final training outcome. Breeding, dogs selected as breeding stock; Qualified, dogs that qualified as working guide dogs; W-Beh, dogs withdrawn for behavioural reasons; W-Health, dogs withdrawn for health reasons; W-Health & Beh, dogs marked as withdrawn for both health and behavioural reasons.

New z-scores were calculated for all dogs (including the 837 previously used in the development phase) based upon means and standard deviations from the entire dataset of 5, 8 and 12 month dogs (n = 1,385, comprising the 837 Q/W-beh dogs used in the development phase plus 548 previously unused dogs), we were able to deduce which dogs would have been flagged in puppy walking, had the system been implemented. Looking at the number of flags given out at each age, the majority of green flags were assigned to qualified dogs (Fig 3). At 8 and 12 months, the majority of red flags were assigned to dogs that were withdrawn for behaviour (Fig 4), and yellow flags were assigned to dogs that qualified or were withdrawn for behaviour at a 1:1 ratio (Fig 5). Red and yellow flags from 5-month scores performed less well at flagging withdrawn dogs. For this reason we would recommend that the thresholds for assigning green flags can be applied to scores collected from 5 months of age, but that thresholds for assigning red and yellow flags only be applied to scores given at 8 and 12 months of age.

**Fig 6:**
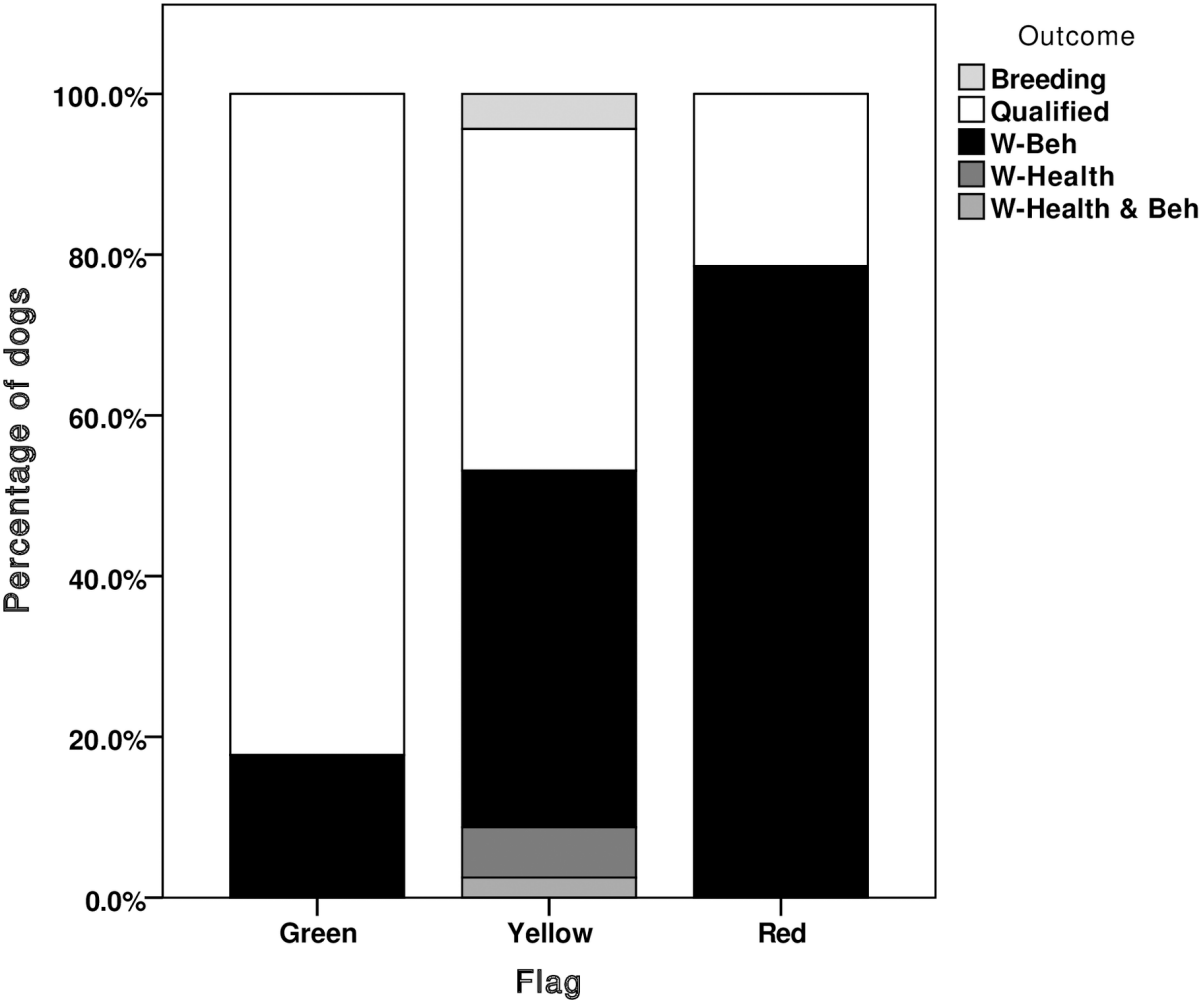
Stacked bar chart showing the percentage of dogs (broken down by training outcome) that would have received either a green, yellow or red flag had the final system been implemented. Green flags were assigned using the 5, 8 and 12-month data, whilst red and yellow flags were assigned using the 8 and 12-month data only. Breeding, dogs selected as breeding stock; Qualified, dogs that qualified as working guide dogs; W-Beh, dogs withdrawn for behavioural reasons; W-Health, dogs withdrawn for health reasons; W-Health & Beh, dogs marked as withdrawn for both health and behavioural reasons.

The predictive ability of the recommended thresholds, taken as a whole, (in terms of numbers of individual dogs that would have received one or more of each flag), were good (Fig 6). Using all three assessments (5, 8 and 12 months) to allocate ‘green’ flags identified 69 qualified dogs and 12 dogs withdrawn for behaviour, giving a green positive predictive value (PPV) of 8.4% and a sensitivity of 85%. From the 8 and 12-month assessments, 32 withdrawn dogs were red flagged and 10 qualified, achieving PPV of 8.4% and a sensitivity of 83% for red flag allocation. A total of 161 dogs received at least one yellow flag for a trait on either the 8 or 12-month assessment. Of these, 68 qualified and 72 were withdrawn for behaviour, successfully achieving a 1:1 ratio of qualified to withdrawn dogs.

### Evaluation—Temporal consistency

Dogs were consistently ranked on all scales considered for each pairing of ages (using Spearman’s correlations) and across all three age groups (Table 3). Correlations ranged from a minimum of 0.42 (General Anxiety 5M-12M) to a maximum of 0.69 (Trainability 8M-12M). As would be expected, correlations were lowest between 5M-12M of age, with a mean *rho* of 0.48, compared to 0.58 for 5M-8M and 0.63 for 8M-12M.

### Evaluation—Construct validity

The majority (13/16) of predicted correlations had coefficients of >0.40 (S7 Table). All correlations were in the predicted direction. The mean correlation strength (after removing valence) was 0.43.

### Evaluation—Concurrent criterion validity

Two of the three PCA’s on the juvenile behaviour test variables achieved acceptable diagnostics and were able to reduce 17 variables down to 6 and 7 components scores for comparison against the 5 and 8-month scales, respectively (see S8 and S9 Tables for PCA loadings). The third PCA, for behavioural ‘events’ recorded throughout all subtests (whines, barks, yawns, scratches, lip-licks and jumps), failed to achieve KMO statistic of more than 0.6, so the decision was taken to analyse these variables separately.

Significant predicted associations between test responses and PTSQ scores were identified for all traits, for at least one age, excepting Body Sensitivity (see S3 Table for all predicted associations and results). Excitability and Distractibility converged best, with significant associations at both ages. For Excitability, 33% (1/3) of predicted associations were present at 5M and 66% (2/3) at 8M, whilst 33% were present for Distractibility at 5M (3/9) and 50% at 8M (4/8). Trainability converged with test measures only at 8 months of age with 57% of predicted associations present at this age (4/7). For Adaptability, 25% of predictions (2/8) were significant at 5M and none at 8M, however, one of these (number of scratches) was related to the score in the opposite way than predicted; positively instead of negatively. For General Anxiety only one prediction was significant and this was ‘low’ greeting posture, which was positively associated with the score at 8 months of age (1/7).

## Discussion

The main aim of this study was to design a questionnaire-style method of assessing personality in juvenile guide dogs that is feasible for regular application on large scales. To achieve this aim three objectives were set: (1) to develop a questionnaire based on identifying groups of correlated question items (scales); (2) to evaluate the questionnaire for use as a predictive decision assistance model for Guide Dogs; 3) to evaluate the questionnaire for temporal consistency, construct validity and concurrent criterion validity, by comparing scale scores of dogs to their responses in a concurrent practical behaviour test. All three objectives were met. Seven scales of grouped correlated questions were identified based on optimum internal reliability and named: Adaptability; Body Sensitivity; Distractibility; Excitability; General Anxiety; Trainability and Stair Anxiety. All of these demonstrate the potential to predict later training outcome for individual dogs by flagging dogs based on threshold values on the scales, corrected for the population average. There was good support for the temporal consistency, construct validity and concurrent validity of the questionnaire and this is believed to be the first behavioural assessment questionnaire which has been shown to have the ability to predict training outcome for individual dogs most and least suitable to the guiding role.

### Development

The development of the questionnaire was informed by surveys of Guide Dogs training staff and the main reasons assigned for behavioural withdrawal of a dog from the training program. To meet the scientific requirements of assessing dog personality, items were selected from previous studies where possible. Where required, new items were made to suit the requirements of the population. Many items could potentially have been sourced from the C-BARQ [29]; a reliable, valid and most widely used trait rating of dog personality [29–32]. However, some C-BARQ items were more relevant to pet dogs, than juvenile guide dogs. Furthermore, for copyright and cross-study comparisons it was not appropriate to alter items within any C-BARQ scales. No entire C-BARQ scale was applicable to this study; therefore due to copyright restrictions we were unable to use any C-BARQ items.

The seven scales that emerged from this study achieved high levels of internal reliability, at all ages, with the exception of Body Sensitivity, which was only just within the acceptable limit for internal reliability (alpha of >0.5). Part of the definition of animal personality is that behavioural responses show consistency between similar situations [33–35]. The scales General Anxiety, Excitability and Distractibility were all comprised of question items that described the same behaviour in different situations or in response to different stimuli. The high level of internal reliability for these scales could be suggestive of situational consistency, which lends support to these scales being measures of personality traits.

### Predictive criteron validity

All scales showed statistical associations with guide dog training outcome consistent at all three ages, except for Distractibility, which was only significantly related to training outcome when dogs were 12 months of age. All relationships were as expected, with high scores for Trainability and Adaptability being related to increased chances of qualification whilst high scores for General Anxiety, Excitability, Distractibility, Stair Anxiety and Body Sensitivity were associated with reduced chances of qualification and increased odds of withdrawal from the program. The six scales that showed the greatest sensitivity to training outcome (in terms of odds ratio impact) were: 8M General Anxiety and Body Sensitivity; 12M General Anxiety, Body Sensitivity, Adaptability and Trainability. Although the odds ratios are close to 1 the use of the continuous scale means that the odds of a dog being withdrawn increased or decreased with every 1mm on the scale. For example, each 1mm increase in ratings for General Anxiety at 8 and 12 months, was respectively associated with a 2.3% and 2.7% increase in odds of being withdrawn for behavioural reasons. Trainability and Adaptability were both negatively associated with withdrawal, and every 1mm increase in their scores at 12 months was associated with reduced odds of withdrawal by 2.2% and 2.3%, respectively.

### Creating a predictive model

To allow for implementation and business use, a decision assistance model for predicting success in guide dog training was created using threshold values in scale z-scores to flag dogs as likely to qualify or be withdrawn for behavioural reasons. The predictive ability of the model was weakest at 5 months of age with only dogs most likely to qualify able to be reliably detected by scores given at this age. Due presumably to the heterogeneous nature of the behaviour of dogs withdrawn from training, the trait scores did not form multivariate models and each score, at each age, thus represents a separate predictive model. This means that each dog has the potential to receive multiple flags, for multiple traits, and whilst a dog may score well for some traits it could score poorly on others.

Overall, sensitivity was high, above 83% for all red and green flags, indicating good target discrimination, and the PPV for both flags was above 8.4%. A small proportion of dogs (10) received red flags for Excitability or General Anxiety but went on to qualify. It is possible that these dogs were individuals with behaviour issues that received successful training or management interventions; alternatively these behaviour issues may recur once outside of the training program. Additionally, 12 dogs with green flags were later withdrawn from the program. Due to the large impact of environment on dog-behavioural development [36] we would hypothesis that after their assessments these dogs may have had a life-experience that altered their behaviour (for example a dog-attack, see [37,38]) or that they had singularly good scores for one trait and poor scores for others that led them to have an overall profile unsuitable to guiding work. Due to the heterogeneous nature of withdrawal reasons, this analysis considered each trait scale separately, but it is hoped that when in use by Guide Dogs that a dog’s behaviour profile across all traits could be considered on a case-by-case basis. Follow-up of these dogs through their working life, and investigation into their progress and handing during training, is recommended to evaluate the factors involved in their qualification and the quality of the dog-owner unit in the long term.

In addition, it could be useful to extend follow-up into working life. This would contribute to knowledge of why some working dogs are retired early for behavioural reasons (e.g.[39] and the human and dog factors which contribute to a dog-owner pairing working successfully or not.

Yellow flags assigned to dogs at 8 and 12 months of age identified 161 ‘at risk’ dogs. Looking at just the dogs that qualified or were withdrawn for behaviour (140) 51% of these dogs were withdrawn and 49% qualified; this represents a 1:1 ratio as compared to the typical ratio of 1:3 for dogs withdrawn for behaviour to dogs that qualify. Yellow flagged dogs show behaviour that makes them more vulnerable to withdrawal, but clearly under certain circumstances these dogs can qualify. Dogs that receive yellow flags for one or more traits could be more closely monitored, with additional training or environment interventions implemented to try and reduce the chances of withdrawal.

Despite the comparatively large sample size utilised in this study, due to the unbalanced data it was not deemed amenable to cross-validation. Any partition of the dataset would have not allowed a sufficiently large sample size for robust analysis. For these reasons the flag system proposed in this study cannot be considered to be cross-validated, despite the otherwise rigorous evaluation of reliability and validity. It is recommended that Guide Dogs check the performance of the system on an annual basis and adjust the flag thresholds accordingly; such a self-regulating system would allow the organisation to adapt to any changes in the populations behavioural profile as a result of breeding or training interventions. It is also recommended that the z-scores for new dogs initially be calculated from mean and standard deviations provided by this population, but that this be recalculated automatically to exclude the oldest dogs in the system as data for new dogs are entered so that the reference population is always current within the organisation.

To our knowledge, this assessment represents the first questionnaire style assessment of working dog behaviour to be published that was able to classify individual dogs according to their likely training outcomes. This behavioural questionnaire has been demonstrated to provide valid information on dog behaviour and so could also be used for purposes other than predicting outcome, for example to inform decisions about training progress, career selection (breeding, guiding or buddy dog) and matching with a Guide Dog owner. The PPV’s here of 8.5% and 8.4% for predicting qualified and withdrawn dogs, respectively, could be considered to be low compared to standardised tests of working dog behaviour in puppies and juveniles, which have been shown to provide PPV’s ranging between 8% in 8-week-old guide dog puppies [5], 33% in 6-month-old trainee police dogs [12] and 87% in 8-month-old juvenile guide dogs [10]. However, standardised behaviour tests are costly to implement and to evaluate in large organisations such as Guide Dogs, where questionnaire based assessments would be comparatively economical and feasible. Even small PPV’s such as these would be useful to Guide Dogs if they allowed the most unsuitable dogs to be removed from training earlier, or improve qualification rates by allowing for successful early interventions. For example, simply focussing on removing the small number of dogs that received red flags we can evaluate how much time could have been saved if they had been withdrawn at the time they were flagged compared to the time they actually were withdrawn. Removing red flagged dogs could have saved a cumulative total of 499 weeks of time in puppy walking, and 258 weeks of time in formal training. Earlier removal of these dogs, though few, would represent a considerable time and therefore cost saving for the organisation. This aspect of the tool is however only a small one, with many other potential benefits to profile dogs using this system such as highlighting dogs that are most likely to qualify and those at risk to allow for tailored interventions; the benefits of which cannot be readily quantified.

### Temporal consistency

Compared to previous studies reviewed in a meta-analysis, there was strong evidence of temporal consistency in the questionnaire scales between 5M, 8M and 12M. Correlations ranged from 0.42 to 0.69, whereas the meta-analysis found mean correlations of 0.34 for puppies and juveniles (<12 months of age) and 0.53 in adult dogs (>12 months of age)[26]. The strength of the correlations from this questionnaire completed by trained dog handlers also compares favourably to similar questionnaires completed by dog carers (correlations 0.25 to 0.56 [31] and 0.18 to 0.66 [10]). The lower end of correlations found here was higher than that found in the questionnaires scored by carers, a result which would be expected if trained dog handlers were more reliable/consistent in their evaluations, although inter-rater reliability was not possible to assess due to there being only one handler per dog. In line with [26], consistency correlations decreased with increasing time intervals, with the lowest correlation being between the first and last assessments (5M-8M, a 26 week gap), although this correlation (0.48) was still above those typically found in dogs less than 12 months of age (0.34).

### Construct validity

All predicted correlations between the scales were in the direction predicted if the scales were measuring the traits they were intended to measure, with the majority (13/16) showing correlations >0.40 (mean correlation of 0.43). These results are in line with previous findings from our group using a similar questionnaire completed by the dogs’ puppy walkers [36] and support the construct validity of the questionnaire scales.

### Concurrent criterion validity

Assessment of the concurrent validity questionnaire utilised a smaller subset of dogs and involved investigating predicted convergent and divergent relationships between questionnaire scales and juvenile behaviour test responses. Validation is notoriously difficult to ascertain by comparing these two assessment methods as the aim is to compare subjective personality scores, gained from observations over time and across situations, against reductionist behavioural responses recorded at one time, in one place [40]. Even though both methods can be equally reliable and informative, they are often found to be measuring different things [41]. However, the results from this study were encouraging with significant, though low, convergence and divergence found between the test responses and scores for Excitability, Distractibility, Trainability and Adaptability. The proportion of convergence ranged from 0%-66% and significant correlations ranged from 0.22–0.57. The only trait which failed to show associations with test measures was Body Sensitivity, and General Anxiety was only significantly associated to one test measure at one age. Overall, convergence between the two methods was better at 8 months of age than 5, perhaps due to increased familiarity of the puppy training supervisors by 8 months of age or an increase in the stability of behaviour over time [9].

Distraction in guide dogs represents 24% of behavioural withdrawals within Guide Dogs, UK and has been reported to be of importance in withdrawals within other guide dog agencies [15]. However, no questionnaire assessment has been published that reliably and validly assesses distraction-related behaviour in juvenile dogs. The Distractibility scale was more strongly associated with obedience responses in the test, than it was with the responses to the distraction stimuli, suggesting that the Distractibility scale and the distraction subtest measures are assessing different facets of behaviour. The validity of the Excitability scale was supported by positive convergent associations with jumping and playing in the behaviour test. Jumping and playing were consistent between individuals across the tests [10]. Trainability converged with obedience responses at 8 months, but not 5 months of age, which could be explained by obedience responses being temporally inconsistent between 5 and 8 months [10] or the dogs having less experience of obedience at 5 months. There was only one association that occurred in a direction opposite to that expected; scratching, which was positively associated with Adaptability at 5 months of age (and approaching significance at 8 months). This apparent discriminant ‘error’ could be explained by the fact that scratching, which is associated with anxiety or internal conflict in dogs [42], may in fact relieve the experience of stress by acting as an active coping mechanism.

The low agreement between General Anxiety scores and test responses could be explained by the fact the test was designed to limit any anxiety for dogs involved [10]. There were subtests (body check, tea towel and head ring) designed to measure body sensitivity, however these did not show convergence with the Body Sensitivity scale in the questionnaire. Of seven studies that compare questionnaire scores of dog behaviour to test responses [43–49], only two studies were comparing questionnaire scores to discrete behavioural codings [47,50]. The number and strength of predicted associations found here compare favourably to those two studies. It has previously been suggested that ‘rater coding’ methods (where observers score dogs’ behaviour on scoring scales based on behavioural descriptions) would be more suitable for scoring behavioural tests than individual behavioural codings [48,51], and this could be considered a limitation of this study. Whilst behavioural coding is often considered more objective, and can help to elucidate the potential function of individual behaviour, it can fail to take account of the complex temporal or concurrent relationships between different behaviour or contexts [18,52].

## Conclusions

A questionnaire was created for completion by the dog’s puppy training supervisor (PTS) that contained 39 items, scored with a visual analogue scale. These items formed seven aggregate scales named: Adaptability; Body Sensitivity; Distractibility; Excitability; General Anxiety; Trainability and Stair Anxiety. Rigorous evaluation of the questionnaires reliability and validity leads us to conclude that the scales Distractibility, Excitability, General Anxiety, Trainability and Adaptability could be considered to be measuring personality traits in juvenile guide dogs, whilst all scales showed evidence of predictive validity when compared to Guide Dogs training outcome. Predictive validity was evidenced for all scales from as early as 5 months, and combined with the creation of a novel ‘flag’ system to aid decision-making, scores given to dogs from 5 months of age were able to identify those most likely to qualify whilst scores at 8 and 12 months of age could identify dogs at high risk of withdrawal. Such evidence of predictive ability of a questionnaire assessment has not been previously presented.

This questionnaire, designed to completion by training supervisors of juvenile guide dogs, and the associated decision assistance model created here, has shown great promise for use in identifying dogs most likely to qualify and those at greatest risk of withdrawal from the training program, earlier than they are currently being identified. If implemented, such a model could be of great value for reducing time spent in training for dogs most unsuited to guiding work and allow for ‘at risk’ dogs to be allocated to bespoke training schemes or interventions to try to improve their chances of success.

## Supporting information

S1 Table Subtests and behavioural coding measures from a juvenile guide dog behaviour test used for comparison against the PTSQ scale scores (adapted from Harvey et al., 2016a). (DOCX)

S2 Table The original 39 puppy training supervisor questionnaire (PTSQ) items, ordered according to the trait they were designed to represent, not in order of appearance in the questionnaire. On each new page the text “This dog…” appeared as a prefix to each item. *S*uperscript numbers provide reference to the origin of the item: ^1^ Serpell & Hsu (2001); ^2^ Arata et al (2010); ^3^ Vas et al (2007); ^4^ Guide Dogs PW survey; ^5^ new item; ^6^ altered or created following PTS panel feedback; ^7^ Goddard & Beilharz (1983).
(DOCX)

S3 Table PCA loadings from each age at assessment (5, 8 and 12 months) for 38* of the items from the PTSQ. Results given are component loadings based upon varimax rotation, with loadings below 0.4 suppressed. Items are ordered according to the groups they were designed for and expected to form. Those highlighted represent five groupings of items that emergered together as groups consistenctly from every one of the three PCA's. A key to the names of the highlighted groups can be found below. * the 39th item was only asked when dogs were 12 months of age so was not included in the PCA's and was treated as a miscellaneous item.
(DOCX)

S4 Table The 39 PTSQ items ordered as they were following Initial Refinement and shown with p-values from univariate logistic regressions against training outcome (5M n = 837, 8M n = 832, 12M n = 811). (PDF)

S5 Table Types of Z-score, at each age, which showed best predictive ability in terms of identifying individual dogs. NF, no flag able to be assigned. Note: Yellow flags were based on red flag Z-scores except where red flags could not be assigned when they were instead based on green flag Z-scores.
(DOCX)

S6 Table *A priori* predicted correlations between the final scales of the puppy training supervisor questionnaire. (DOCX)

S7 Table Rotated component matrix loadings for the responses to subtest 7 (tea towel) from the juvenile guide dog behaviour test, at 5 and 8 months of age. The PCA’s achieved KMO statistics of 0.77 and 0.72 for the 5 and 8-month tests, respectively, with Bartlett’s test of spherictiy significant to p<0.001 for both. Cumulative variance explained by the components was 61.5% at 5 months and 58.7% and 8 months.
(DOCX)

S8 Table Rotated component matrix loadings for the responses to subtests 8 (food), 9 (robin), 10 (pigeons) and 11 (human) from the juvenile guide dog behaviour test, at 5 and 8 months of age. The PCA’s achieved KMO statistics of 0.60 and 0.65 for the 5 and 8-month tests, respectively, with Bartlett’s test of spherictiy significant to p<0.001 for both. Cumulative variance explained by the components was 75.0% at 5 months and 63.2% and 8 months.
(DOCX)

S9 Table A list of all predicted associations between puppy test behavioural measures and puppy training supervisor questionnaire (PTSQ) scales. Coef., test coefficients; these are correlation coefficients (rho) for all continuous, component or mean data and standardised test statistics for all tests with binary data from Mann-Whitney U tests (shown in italics). Significant associations are highlighted in bold. Associations in the direction opposite that predicted are shown in bold italics. * p<0.05, **p<0.01, ***p<0.001.
(DOCX)

S10 Table (XLSX)
