# Supplementary material for: An evidence-based decision assistance model for predicting training outcome in juvenile guide dogs
Source: PLoS One. 2017 Jun 14;12(6):e0174261. doi: 10.1371/journal.pone.0174261 (PMC5470660; doi:10.1371/journal.pone.0174261)
Supplement: S1 Table — (DOCX) [file pone.0174261.s001.docx]

Supplementary table 1. Subtests and behavioural coding measures from a juvenile guide dog behaviour test used for comparison against the PTSQ scale scores (adapted from Harvey et al., 2016a).

| **Subtest** | **Behaviour** | **Type** | **Definition** |
| --- | --- | --- | --- |
| All | Jumps | Continuous (count) | Dogs front two, or more, paws off the ground simultaneously (but not when rearing due to strong lead pulling) |
|  | Whines | Continuous (count) | Frequency of whining bouts, with a bout defined as: a continuous emission of whining ending when whining stops |
|  | Scratches | Continuous (count) | Dog scratches itself with back feet |
|  | Barks | Binary | Scored as whether a bark was observed at any point during the video scoring |
|  | Shakes | Binary | Shakes head or whole body (in subtests 5-7 only) |
|  | Lip-licks | Frequency | Tongue briefly seen outside of mouth, sweeping across lips/muzzle or up to nose |
| Subtest 1- Meet a Stranger | Low Posture | Binary (1/0) | Low posture during greeting: front legs bent; tail neutral or low and wagging; head lowered and ears backwards |
| Subtest 2 and 3 – PW and stranger (STR) obedience | Sit/Wait/Down Response | Categorical | Dog obeyed 'sit' ‘wait’ or ‘down’ command and sits on hind quarters in response to (1) first command; (2) second command or more; (3) does not respond to command appropriately |
|  | Gaze Proportion | Continuous (%) | The proportion of time spent gazing at the face of the handler, relative to the total length of the subtest |
|  |  |  |  |
| Subtest 5- Body Check | Mouths | Continuous (count) | Low pressure, non-injurious grab of testers limbs or clothes with mouth. Recorded as a count of total number observed |
| Subtest 6 - Head Ring | Ear Position | Binary (1/0) | Neutral - individual relaxed ear state, neither forwards nor backwards facing; Backwards - ears flattened backwards against the head, exposing the inner ear lining to view |
|  | Tail Height | Categorical | Neutral - relaxed tail allowed to fall vertically from where the tail joins the spine; Half Up - he tail falling below the level of the dogs back, but raised from neutral; Up - tail in line with, or above, the level of the dogs back |
|  | Body Posture | Binary (1/0) | Neutral- weight evenly distributed, head not extended; or Stretched- weight over front legs and head extended, when head inserted in ring |
| Subtest 7 –  Tea-Towel | Attempts to Remove | Binary (1/0) | An attempt by the dog, successful or not, to remove the tea-towel from their back |
|  | Turns Head | Binary (1/0) | Head turned to look at the tea-towel with no attempt to remove |
|  | Change from Neutral | Binary (1/0) | Dogs’ body posture changed from neutral when tea-towel placed on back. Changes included: arched back; lowered tail and backwards ears |
|  | Plays with | Binary (1/0) | Dog played with the tea-towel after removal. Play included: shaking; tearing at or running with the tea-towel held in mouth |
| Subtest 8 - Subtest 10:  Food; Robin & Pigeon Distractions | Pull Strength | Categorical | None – lead may be tense but dogs is weight evenly distributed across all four feet and no straining against the lead; Medium – head extended towards stimulus, weight pushing forwards and straining against the lead, all paws remain on the ground; Strong –weight forwards, the dog is straining against the lead with head extended towards stimulus, back legs are stretched and one or more front paws raised off the floor |
|  | Time Oriented | Continuous (seconds) | Time the dog remained oriented towards the stimulus, with head or head & body, after first recall prompt |
|  | Approaches | Binary (1/0) | Dog left side of Experimenter and walked towards the stimulus |
| Subtest 11: Human Distraction | Pull Strength (greet) | Categorical | As above |
|  | Jumps | Binary (1/0) | Jumped up with front paws placed on human distraction (experimenter 3) |
