# Supplementary material for: An evidence-based decision assistance model for predicting training outcome in juvenile guide dogs
Source: PLoS One. 2017 Jun 14;12(6):e0174261. doi: 10.1371/journal.pone.0174261 (PMC5470660; doi:10.1371/journal.pone.0174261)
Supplement: S2 Table — On each new page the text “This dog…” appeared as a prefix to each item. Superscript numbers provide reference to the origin of the item: 1 Serpell & Hsu (2001); 2 Arata et al (2010); 3 Vas et al (2007); 4 Guide Dogs PW survey; 5 new item; 6 altered or created following PTS panel feedback; 7 Goddard & Beilharz (1983). (DOCX) [file pone.0174261.s002.docx]

Supplementary Table 2. The original 39 puppy training supervisor questionnaire (PTSQ) items, ordered according to the trait they were designed to represent, not in order of appearance in the questionnaire. On each new page the text “This dog…” appeared as a prefix to each item. *S*uperscript numbers provide reference to the origin of the item: ^1^ Serpell & Hsu (2001); ^2^ Arata et al (2010); ^3^ Vas et al (2007); ^4^ Guide Dogs PW survey; ^5^ new item; ^6^ altered or created following PTS panel feedback; ^7^ Goddard & Beilharz (1983).

| **Item** | |
| --- | --- |
| ***Excitability*** |  |
| Is calm and quiet ^5^ | |
| Exhibits a high degree of excitement (jumps up; barks; coughs etc.) when goes somewhere new ^5^ | |
| Is initially excitable (jumps up; barks; coughs etc.), but quickly settles ^2^ | |
| Exhibits a high degree of excitement (jumps up; barks; coughs etc.) when you initially enter the home ^5^ | |
| ***Anxiety*** | |
| Is obviously startled by loud or unexpected sounds ^1, 6^ | |
| Is obviously startled by odd or unexpected things or objects ^1, 6^ | |
| Is anxious or uneasy in new situations ^1, 6^ | |
| Backs away from or is reluctant to pass objects on the street (such as collecting boxes, bin bags or children's ride-on toys) ^4^ | |
| Appears uneasy on closed stairs ^5, 6^ | |
| Appears uneasy on open or unusual (e.g. glass) stairs ^5, 6^ | |
| Adapts well to new situations and environments ^5; 6^ | |
| Recovers quickly after being unsettled or frightened ^5; 6^ | |
| ***Body Sensitivity*** | |
| Is uneasy with being physically handled/groomed ^5^ | |
| Appears uneasy or uncomfortable when putting on Guide Dog equipment (including collars) ^5^ | |
| Shows a rapid response to correction by handling ^7^ | |
| Is reluctant to walk close to the handler ^5^ | |
| ***Distractibility*** | |
| Pulls (including lunging) towards unfamiliar dogs ^5^ | |
| Pulls towards/distracted by food on the ground or food scents ^2; 6^ | |
| Shows interest (attempts to greet , sniffs, wags tail) when directly approached by children or member of the public ^5^ | |
| Shows interest (attempts to greet , sniffs, wags tail) when passing children or members of the public ^5^ | |
| Shows interest (attempts to greet , sniffs, wags tail) when it encounters other dogs ^5^ | |
| Attempts to sniff objects in the street ^5; 6^ | |
| ***Attentiveness*** | |
| Is attentive to you ^5^ | |
| Attention can be attracted easily but it loses interest soon ^3, 6^ | |
| Will look at you when you talk to it directly in the home environment ^5; 6^ | |
| Attention can be easily distracted ^3^ | |
| ***Trainability*** | |
| Seems not to listen even if it knows someone is speaking to it ^3^ | |
| Needs obedience commands repeating to get a response ^4^ | |
| ‘Stays/waits’ when instructed to ^3; 6^ | |
| Refuses to obey commands, which in the past it has proven it has learned ^3, 6^ | |
| Responds immediately to the recall command when off lead ^5^ | |
| Readily accepts the responsibility of decision making (12 months only) ^5^ | |
| Is easy to control ^5; 6^ | |
| Is eager to please ^5; 6^ | |
| ***Immaturity*** | |
| Is active and energetic ^1^ | |
| Is mischievous ^5^ | |
| ***Miscellaneous*** | |
| Requires an indoor kennel when left alone ^4;6^ | |
| Is stubborn ^5; 6^ | |
| Is friendly ^5; 6^ | |
