# Supplementary material for: An evidence-based decision assistance model for predicting training outcome in juvenile guide dogs
Source: PLoS One. 2017 Jun 14;12(6):e0174261. doi: 10.1371/journal.pone.0174261 (PMC5470660; doi:10.1371/journal.pone.0174261)
Supplement: S3 Table — Results given are component loadings based upon varimax rotation, with loadings below 0.4 suppressed. Items are ordered according to the groups they were designed for and expected to form. Those highlighted represent five groupings of items that emergered together as groups consistenctly from every one of the three PCA's. A key to the names of the highlighted groups can be found below. * the 39th item was only asked when dogs were 12 months of age so was not included in the PCA's and was treated as a miscellaneous item. (DOCX) [file pone.0174261.s003.docx]

**Supplementary Table 3.**  A list of all predicted associations between puppy test behavioural measures and puppy training supervisor questionnaire (PTSQ) scales. Coef., test coefficients; these are correlation coefficients (*rho*) for all continuous, component or mean data and standardised test statistics for all tests with binary data from Mann-Whitney U tests (shown in *italics*). Significant associations are highlighted in **bold**. Associations in the direction opposite that predicted are shown in ***bold italics***. * p<0.05, **p<0.01, ***p<0.001

| **PTSQ scale** | **Puppy test subtest** | **Behaviour/Measure** | **Type** | **Test** | **Predicted relationship** | **5M Coef.** | **8M Coef.** |
| --- | --- | --- | --- | --- | --- | --- | --- |
| Excitability | All | Jumps | Continuous (count) | Spearman’s Rank | Positive | **0.36**** | **0.57***** |
|  | Subtest 7 – Tea-Towel | Plays/Removes | Component score | Spearman’s Rank | Positive | 0.12 | **0.24*** |
|  | Subtest 11 - Human | Human response | Component score | Spearman’s Rank | Positive | 0.04 | 0.17 |
| General Anxiety | All | Lip-licks | Continuous (count) | Spearman’s Rank | Positive | 0.11 | 0.09 |
|  |  | Whines | Continuous (count) | Spearman’s Rank | Positive | -0.09 | 0.14 |
|  |  | Yawns | Continuous (count) | Spearman’s Rank | Positive | -0.127 | 0.07 |
|  |  | Scratches | Continuous (count) | Spearman’s Rank | Positive | -0.08 | 0.03 |
|  | Subtest 1- Meet a Stranger | Low Posture | Binary (1/0) | Mann-Whitney U | Positive | *0.95* | ***2.01**** |
|  | Subtest 4 - Path | Crossed | Binary (1/0) | Mann-Whitney U | Negative | NA | *-0.02* |
|  | Subtest 6 - Head Ring | Head Ring Score | Continuous | Spearman’s Rank | Positive | 0.16 | 0.17 |
|  | Subtest 7 – Tea-Towel | Changes/Turns | Component score | Spearman’s Rank | Positive | -0.17 | -0.03 |
|  | Subtests 5-7 | Shakes | Binary (1/0) | Mann-Whitney U | Negative | *-0.15* | *-0.83* |
| Distractibility | All | Jumps | Continuous (count) | Spearman’s Rank | Positive | **0.28*** | **0.40***** |
|  | Subtest 2 - PW Obedience | PW Obedience Response | Mean | Spearman’s Rank | Positive | **0.25*** | **0.25*** |
|  |  | Gaze Proportion | Continuous (%) | Spearman’s Rank | Negative | -0.13 | **-0.24*** |
|  | Subtest 3 - STR Obedience | STR Obedience Response | Mean | Spearman’s Rank | Positive | 0.003 | **0.22*** |
|  |  | Gaze Proportion | Continuous (%) | Spearman’s Rank | Negative | -0.06 | -0.13 |
|  | Subtest 8 - Food | Food response | Component score | Spearman’s Rank | Positive | **0.27*** | -0.05 |
|  | Subtests 9 & 10 - Pigeon | Bird distraction 8M | Component score | Spearman’s Rank | Positive | NA | 0.16 |
|  |  | Pigeons approach 5M | Component score | Spearman’s Rank | Positive | 0.09 | NA |
|  |  | Bird distraction 5M | Component score | Spearman’s Rank | Positive | 0.02 | NA |
|  | Subtest 11 - Human | Human response | Component score | Spearman’s Rank | Positive | -0.70 | 0.06 |
| Trainability | All | Jumps | Continuous (count) | Spearman’s Rank | Negative | -0.17 | **-0.38***** |
|  | Subtest 2 - PW Obedience | PW Obedience Response | Mean | Spearman’s Rank | Negative | -0.15 | **-0.38***** |
|  |  | Gaze Proportion | Categorical | Spearman’s Rank | Positive | 0.17 | **0.42***** |
|  | Subtest 3 - STR Obedience | STR Obedience Response | Mean | Spearman’s Rank | Negative | -0.14 | -0.20 |
|  |  | Gaze Proportion | Continuous (%) | Spearman’s Rank | Positive | 0.12 | 0.20 |
|  | Subtest 8 - Food | Food response | Component score | Spearman’s Rank | Negative | -0.11 | -0.12 |
|  | Subtests 9 & 10 - Pigeon | Bird distraction 8M | Component score | Spearman’s Rank | Negative | NA | **-0.22*** |
|  |  | Pigeons approach 5M | Component score | Spearman’s Rank | Positive | -0.22 | NA |
|  |  | Bird distraction 5M | Component score | Spearman’s Rank | Positive | -0.07 | NA |
| Body Sensitivity | Subtest 5 - Body Check | Mouths | Continuous (count) | Spearman’s Rank | Positive | 0.07 | -0.06 |
|  | Subtests 5-7 | Shakes | Binary (1/0) | Mann-Whitney U | Negative | *-1.57* | *-1.35* |
|  | Subtest 6 - Head Ring | Head Ring Score | Continuous | Spearman’s Rank | Positive | 0.10 | 0.08 |
|  | Subtest 7 – Tea-Towel | Changes/Turns | Component score | Spearman’s Rank | Positive | -0.12 | 0.05 |
| Adaptability | All | Lip-licks | Continuous (count) | Spearman’s Rank | Negative | -0.07 | -0.12 |
|  |  | Whines | Continuous (count) | Spearman’s Rank | Negative | 0.06 | -0.08 |
|  |  | Barks | Binary (1/0) | Mann-Whitney U | Negative | 0.40 | *1.05* |
|  |  | Yawns | Continuous (count) | Spearman’s Rank | Negative | **-0.24*** | 0.16 |
|  |  | Scratches | Continuous (count) | Spearman’s Rank | Negative | ***0.23**** | 0.18 |
|  | Subtest 4 - Path | Crossed | Binary (1/0) | Mann-Whitney U | Positive | NA | *0.30* |
|  | Subtest 6 - Head Ring | Head Ring Score | Continuous | Spearman’s Rank | Negative | -0.05 | -0.04 |
|  | Subtest 7 – Tea-Towel | Changes/Turns | Component score | Spearman’s Rank | Negative | 0.17 | -0.11 |
|  | Subtests 5-7 | Shakes | Binary (1/0) | Mann-Whitney U | Positive | *1.18* | *0.03* |
