# Supplementary material for: An evidence-based decision assistance model for predicting training outcome in juvenile guide dogs
Source: PLoS One. 2017 Jun 14;12(6):e0174261. doi: 10.1371/journal.pone.0174261 (PMC5470660; doi:10.1371/journal.pone.0174261)
Supplement: S4 Table — (PDF) [file pone.0174261.s004.pdf]

| | | 5M Components | | | | | | | | | | 8M Components | | | | | | | | | 12M Components | | | | | | | | | |
|---|---|---|---|---|---|---|---|---|---|---|---|---|---|---|---|---|---|---|---|---|---|---|---|---|---|---|---|---|---|---|
| **Intended dimension** | **Item Wording** | 1 | 2 | 3 | 4 | 5 | 6 | 7 | 8 | 9 | 10 | 1 | 2 | 3 | 4 | 5 | 6 | 7 | 8 | 9 | 1 | 2 | 3 | 4 | 5 | 6 | 7 | 8 | 9 | 10 |
| Anxiety | Is obviously startled by loud or unexpected sounds | | | **0.78** | | | | | | | | | | **0.76** | | | | | | | | | **0.74** | | | | | | | |
| | Is obviously startled by odd or unexpected things or objects | | | **0.78** | | | | | | | | | | **0.84** | | | | | | | | | **0.81** | | | | | | | |
| | Is anxious or uneasy in new situations | | | **0.69** | | | | | | | | | | **0.74** | | | | | | | | | **0.76** | | | | | | | |
| | Backs away from or is reluctant to pass objects on the street (such as collecting boxes, bin bags or children's ride-on toys) | | | **0.66** | | | | | | | | | | **0.76** | | | | | | | | | **0.67** | | | | | | | |
| | Appears uneasy on closed stairs | | | | | | | | 0.80 | | | | | | | | | | 0.83 | | | | | | | | | | **0.87** | |
| | Appears uneasy on open or unusual (e.g. glass) stairs | | | | | | | | 0.72 | | | | | | | | | | 0.74 | | | | | | | | | | **0.83** | |
| Adaptability | Adapts well to new situations and environments | | 0.54 | **-0.52** | | | | | | | | | 0.55 | **-0.54** | | | | | | | | | **-0.63** | | | | | | | |
| | Recovers quickly after being unsettled or frightened | | 0.49 | **-0.49** | | | | | | | | | 0.40 | **-0.56** | | | | | | | 0.51 | | **-0.58** | | | | | | | |
| Attentiveness | Attention can be attracted easily but it loses interest soon | 0.46 | | | | | | | | | | 0.62 | | | | | | | | | | 0.67 | | | | | | | | |
| | Is attentive to you | | 0.72 | | | | | | | | | | 0.74 | | | | | | | | 0.77 | | | | | | | | | |
| | Will look at you when you talk to it directly in the home environment | | 0.48 | | | | | | | | | | 0.64 | | | | | | | | 0.67 | | | | | | | | | |
| | Attention can be easily distracted | 0.56 | | | | | | | | -0.43 | | 0.62 | | | | | | | | | | 0.62 | | | | | | | | |
| Body Sensitivity | Is uneasy with being physically handled/groomed | | | | | | | 0.76 | | | | | | | | | | 0.60 | | | | | | | | | **0.70** | | | |
| | Appears uneasy or uncomfortable when putting on Guide Dog equipment (including collars) | | | | | | | 0.57 | | | | | | | | | | 0.53 | | | | | | | | | **0.60** | | | |
| | Shows a rapid response to correction by handling | | 0.55 | | | | | | | | | | 0.67 | | | | | | | | 0.65 | | | | | | | | | |
| | Is reluctant to walk close to the handler | | | | | | | 0.42 | | | | | | | | | | 0.64 | | | | | | | | | **0.47** | | | |
| Distractibility | Pulls (including lunging) towards unfamiliar dogs | 0.72 | | | | | | | | | | 0.74 | | | | | | | | | | | | **0.58** | | | | | | |
| | Shows interest (attempts to greet , sniffs, wags tail) when directly approached by children or member of the public | 0.79 | | | | | | | | | | 0.59 | | | | | | | | 0.42 | | | | **0.82** | | | | | | |
| | Shows interest (attempts to greet , sniffs, wags tail) when passing children or members of the public | 0.78 | | | | | | | | | | 0.71 | | | | | | | | | | | | **0.78** | | | | | | |
| | Shows interest (attempts to greet , sniffs, wags tail) when it encounters other dogs | 0.73 | | | | | | | | | | 0.71 | | | | | | | | | | | | **0.70** | | | | | | |
| | Pulls towards/distracted by food on the ground or food scents | 0.72 | | | | | | | | | | 0.79 | | | | | | | | | | 0.41 | | **0.55** | | | | | | |
| | Attempts to sniff objects in the street | 0.70 | | | | | | | | | | 0.73 | | | | | | | | | | 0.44 | | **0.49** | | | | | | |
| Excitability | Exhibits a high degree of excitement (jumps up; barks; coughs etc) when goes somewhere new | | | | 0.68 | | | | | | | | | | 0.56 | | | | | | | | | | | 0.73 | | | | |
| | Exhibits a high degree of excitement (jumps up; barks; coughs etc) when you initially enter the home | | | | 0.76 | | | | | | | | | | 0.75 | | | | | | | | | | | 0.66 | | | | |
| | Is calm and quiet | | 0.45 | | -0.61 | | | | | | | | 0.46 | | -0.59 | | | | | | 0.44 | | | | | | | -0.48 | | |
| | Is initially excitable (jumps up; barks; coughs etc), but quickly settles | | | | 0.51 | | | | | | | | | | 0.63 | | | | | | | | | | | 0.72 | | | | |
| Immaturity | Is active and energetic | | | | 0.73 | | | | | | | | | | 0.72 | | | | | | | | | | | | | 0.74 | | |
| | Is mischievous | | | | 0.58 | | | | | | | | | | 0.55 | | 0.53 | | | | | | | | | | | 0.68 | | |
| Trainability | Seems not to listen even if it knows someone is speaking to it | | | | | 0.56 | | | | | | | | | | | 0.54 | 0.46 | | | | 0.58 | | | | | | | | |
| | Refuses to obey commands, which in the past it has proven it has learned | | | | | 0.65 | | | | | | | | | | | 0.63 | | | | | 0.75 | | | | | | | | |
| | Needs obedience commands repeating to get a response | | | | | 0.47 | | | | | | | | | | | 0.48 | | | | | 0.72 | | | | | | | | |
| | 'Stays/waits' when instructed to | | | | | | | | | | 0.70 | | 0.59 | | | | | | | | 0.65 | | | | | | | | | |
| | Is easy to control | | 0.72 | | | | | | | | | | 0.69 | | | | | | | | 0.67 | | | | | | | | | |
| | Is eager to please | | 0.78 | | | | | | | | | | 0.72 | | | | | | | | 0.70 | | | | | | | | | |
| | Responds immediately to the recall command when off lead | | 0.51 | | | | | | | | | | 0.52 | | | | | | | | 0.63 | | | | | | | | | |
| Miscellaneous | Requires an indoor kennel when left alone | | | | | | | | | | 0.54 | | | | | | | | | 0.74 | | | | | | | | | | 0.76 |
| | Is stubborn | | | | | 0.64 | | | | | | | | | | | 0.72 | | | | | 0.69 | | | | | | | | |
| | Is friendly | | 0.60 | | | | | | | | | | 0.62 | | | | | | | | 0.62 | | | | | | | | | |

5M: Extraction Method: Principal Component Analysis.
Rotation Method: Varimax with Kaiser Normalization.a
a Rotation converged in 27 iterations.

8M: Extraction Method: Principal Component Analysis.
Rotation Method: Varimax with Kaiser Normalization.a
a Rotation converged in 8 iterations.

12M: Extraction Method: Principal Component Analysis.
Rotation Method: Varimax with Kaiser Normalization.a
a Rotation converged in 11 iterations.

**Supplementary Table 4.** PCA loadings from each age at assessment (5, 8 and 12 months) for 38* of the items from the PTSQ. Results given are component loadings based upon varimax rotation, with loadings below 0.4 suppressed. Items are ordered according to the groups they were designed for and expected to form. Those highlighted represent five groupings of items that emergered together as groups consistenctly from every one of the three PCA's. A key to the names of the highlighted groups can be found below. * the 39th item was only asked when dogs were 12 months of age so was not included in the PCA's and was treated as a miscellaneous item.

General Anxiety
Stair Anxiety
Adaptability (although these items grouped with General Anxiety at all three ages they were in the opposite direction and also showed some cross-loadings at all ages so we chose to analyse these as a separate group)
Body Sensitivity
Distractibility
