# Supplementary material for: An evidence-based decision assistance model for predicting training outcome in juvenile guide dogs
Source: PLoS One. 2017 Jun 14;12(6):e0174261. doi: 10.1371/journal.pone.0174261 (PMC5470660; doi:10.1371/journal.pone.0174261)
Supplement: S5 Table — NF, no flag able to be assigned. Note: Yellow flags were based on red flag Z-scores except where red flags could not be assigned when they were instead based on green flag Z-scores. (DOCX) [file pone.0174261.s005.docx]

**Supplementary Table 5.** The 39 PTSQ items ordered as they were following Initial Refinement and shown with p-values from univariate logistic regressions against training outcome (5M n=837, 8M n=832, 12M n=811).

| **Scale** | **Item** | **5M** | **8M** | **12M** |
| --- | --- | --- | --- | --- |
| **Trainability** | Attention can be attracted easily but it loses interest soon | 0.019 | 0.027 | <0.001 |
|  | Attention can be easily distracted | 0.008 | 0.112 | 0.001 |
|  | Is stubborn | 0.050 | 0.161 | 0.511 |
|  | Will look at you when you talk to it directly in the home environment | 0.153 | 0.118 | 0.125 |
|  | Seems not to listen even if it knows someone is speaking to it | <0.001 | 0.004 | 0.063 |
|  | Refuses to obey commands, which in the past it has proven it has learned | 0.097 | 0.063 | 0.004 |
|  | Needs obedience commands repeating to get a response | 0.001 | 0.020 | 0.073 |
|  | Is attentive to you | 0.159 | <0.001 | 0.019 |
|  | Shows a rapid response to correction by handling | 0.021 | 0.099 | 0.003 |
|  | Is easy to control | <0.001 | <0.001 | <0.001 |
|  | Is eager to please | 0.002 | <0.001 | 0.001 |
|  | Is friendly | 0.385 | 0.033 | 0.002 |
|  | Stays/waits when instructed to | 0.157 | 0.008 | <0.001 |
|  | Responds immediately to the recall command when off lead | 0.427 | 0.187 | 0.098 |
| **General Anxiety** | Is obviously startled by loud or unexpected sounds | 0.035 | <0.001 | 0.022 |
|  | Is obviously startled by odd or unexpected things or objects | 0.024 | <0.001 | 0.001 |
|  | Is anxious or uneasy in new situations | 0.001 | <0.001 | <0.001 |
|  | Backs away from or is reluctant to pass objects on the street (such as collecting boxes, bin bags or children's ride-on toys) | 0.002 | <0.001 | 0.002 |
| **Adaptability** | Adapts well to new situations and environments | 0.010 | <0.001 | <0.001 |
|  | Recovers quickly after being unsettled or frightened | 0.166 | <0.001 | <0.001 |
| **Excitability** | Exhibits a high degree of excitement (jumps up; barks; coughs etc.) when goes somewhere new | <0.001 | <0.001 | <0.001 |
|  | Exhibits a high degree of excitement (jumps up; barks; coughs etc.) when you initially enter the home | 0.008 | <0.001 | <0.001 |
|  | Is active and energetic | 0.084 | 0.757 | 0.324 |
|  | Is mischievous | <0.001 | 0.008 | 0.007 |
|  | Is calm and quiet | <0.001 | <0.001 | <0.001 |
|  | Is initially excitable (jumps up; barks; coughs etc.), but quickly settles | 0.329 | 0.003 | 0.005 |
| **Body Sensitivity** | Is uneasy with being physically handled/groomed | 0.001 | 0.017 | 0.028 |
|  | Appears uneasy or uncomfortable when putting on Guide Dog equipment (including collars) | 0.007 | 0.004 | 0.002 |
|  | Is reluctant to walk close to the handler | 0.004 | 0.005 | 0.003 |
| **Distractibility** | Pulls (including lunging) towards unfamiliar dogs | 0.007 | 0.017 | <0.001 |
|  | Shows interest (attempts to greet, sniffs, wags tail) when directly approached by children or member of the public | 0.261 | 0.942 | 0.889 |
|  | Shows interest (attempts to greet, sniffs, wags tail) when passing children or members of the public | 0.059 | 0.097 | 0.178 |
|  | Shows interest (attempts to greet, sniffs, wags tail) when it encounters other dogs | 0.296 | 0.325 | 0.032 |
|  | Pulls towards/distracted by food on the ground or food scents | 0.290 | 0.519 | 0.033 |
|  | Attempts to sniff objects in the street | 0.198 | 0.726 | 0.083 |
| **Stair Anxiety** | Appears uneasy on closed stairs | 0.017 | 0.002 | 0.040 |
|  | Appears uneasy on open or unusual (e.g. glass) stairs | 0.002 | <0.001 | 0.053 |
| **Miscellaneous** | Requires an indoor kennel when left alone | 0.206 | 0.043 | <0.001 |
|  | Readily accepts the responsibility of decision making (12M only) | NA | NA | <0.001 |
