# Supplementary material for: An evidence-based decision assistance model for predicting training outcome in juvenile guide dogs
Source: PLoS One. 2017 Jun 14;12(6):e0174261. doi: 10.1371/journal.pone.0174261 (PMC5470660; doi:10.1371/journal.pone.0174261)
Supplement: S6 Table — (DOCX) [file pone.0174261.s006.docx]

**Supplementary Table 6.** Types of Z-score, at each age, which showed best predictive ability in terms of identifying individual dogs. NF, no flag able to be assigned. *Note:* Yellow flags were based on red flag Z-scores except where red flags could not be assigned when they were instead based on green flag Z-scores.

| **Trait** | **5M Green flag Z-score** | **5M Red flag Z-score** | **8M Green flag Z-score** | **8M Red flag Z-score** | **12M Green flag Z-score** | **12M Red flag Z-score** |
| --- | --- | --- | --- | --- | --- | --- |
| Trainability | Within breed & sex | Whole population | Within breed | NF | Within breed & sex | Whole population |
| General Anxiety | Within breed & sex | Whole population | Within breed & sex | Whole population | Within breed & sex | Within breed & sex |
| Adaptability | Within breed & sex | NF | Within breed & sex | Whole population | NF | NF |
| Excitability | Within breed & sex | Whole population | Whole population | Whole population | Within breed & sex | Within breed & sex |
| Body Sensitivity | NF | Whole population | NF | Whole population | NF | Whole population |
| Distractibility | Within breed & sex | Within breed & sex | NF | NF | Whole population | Whole population |
| Stair Anxiety | NF | NF | Within breed & sex | Whole population | NF | Whole population |
