# Supplementary material for: An evidence-based decision assistance model for predicting training outcome in juvenile guide dogs
Source: PLoS One. 2017 Jun 14;12(6):e0174261. doi: 10.1371/journal.pone.0174261 (PMC5470660; doi:10.1371/journal.pone.0174261)
Supplement: S7 Table — The PCA’s achieved KMO statistics of 0.77 and 0.72 for the 5 and 8-month tests, respectively, with Bartlett’s test of spherictiy significant to p<0.001 for both. Cumulative variance explained by the components was 61.5% at 5 months and 58.7% and 8 months. (DOCX) [file pone.0174261.s007.docx]

**Supplementary Table 7.** *A priori* predicted correlations between the final scales of the puppy training supervisor questionnaire.

| \| **Scales** \| **Predicted relationship** \| ***Rho* > 0.4** \| **Correlation coefficient** \| \| --- \| --- \| --- \| --- \| \| Excitability & Trainability \| - \| Yes \| -0.40 \| \| General Anxiety & Trainability \| - \| Yes \| -0.41 \| \| Body Sensitivity & Trainability \| - \| Yes \| -0.55 \| \| Distractibility & Trainability \| - \| Yes \| -0.43 \| \| Stair Anxiety & Trainability \| - \| Yes \| -0.40 \| \| Adaptability & Trainability \| + \| Yes \| 0.32 \| \| Distractibility & General Anxiety \| + \| No \| 0.12 \| \| Body Sensitivity & General Anxiety \| + \| Yes \| 0.55 \| \| Stair Anxiety & General Anxiety \| + \| Yes \| 0.66 \| \| Adaptability & General Anxiety \| - \| Yes \| -0. 62 \| \| Distractibility & Adaptability \| - \| No \| -0.09 \| \| Distractibility & Excitability \| + \| Yes \| 0.46 \| \| Excitability & Adaptability \| - \| No \| -0.12 \| \| Body Sensitivity & Adaptability \| - \| Yes \| -0.57 \| \| Body Sensitivity & Stair Anxiety \| + \| Yes \| 0.62 \| \| Adaptability & Stair Anxiety \| - \| Yes \| -0.49 \| |
| --- | --- | --- | --- | --- | --- | --- | --- | --- | --- | --- | --- | --- | --- | --- | --- | --- | --- | --- | --- | --- | --- | --- | --- | --- | --- | --- | --- | --- | --- | --- | --- | --- | --- | --- | --- | --- | --- | --- | --- | --- | --- | --- | --- | --- | --- | --- | --- | --- | --- | --- | --- | --- | --- | --- | --- | --- | --- | --- | --- | --- | --- | --- | --- | --- | --- | --- | --- | --- |
