# Supplementary material for: An evidence-based decision assistance model for predicting training outcome in juvenile guide dogs
Source: PLoS One. 2017 Jun 14;12(6):e0174261. doi: 10.1371/journal.pone.0174261 (PMC5470660; doi:10.1371/journal.pone.0174261)
Supplement: S8 Table — The PCA’s achieved KMO statistics of 0.60 and 0.65 for the 5 and 8-month tests, respectively, with Bartlett’s test of spherictiy significant to p<0.001 for both. Cumulative variance explained by the components was 75.0% at 5 months and 63.2% and 8 months. (DOCX) [file pone.0174261.s008.docx]

**Supplementary Table 8.** Rotated component matrix loadings for the responses to subtest 7 (tea towel) from the juvenile guide dog behaviour test, at 5 and 8 months of age. The PCA’s achieved KMO statistics of 0.77 and 0.72 for the 5 and 8-month tests, respectively, with Bartlett’s test of spherictiy significant to p<0.001 for both. Cumulative variance explained by the components was 61.5% at 5 months and 58.7% and 8 months.

|  | **5M components** | |  | **8M components** | |
| --- | --- | --- | --- | --- | --- |
| **Variables** | **Plays/Removes** | **Changes/Turns** |  | **Plays/Removes** | **Changes/Turns** |
| Tea towel: 1st Plays with | **0.838** | -0.113 |  | **0.797** | -0.085 |
| Tea towel: 1st Attempts to remove | **0.825** | -0.191 |  | **0.833** | 0.058 |
| Tea towel: 2nd Plays with | **0.817** | -0.076 |  | **0.796** | 0.004 |
| Tea towel: 2nd Attempts to remove | **0.817** | -0.011 |  | **0.836** | -0.033 |
| Tea towel: 1st Turns | **-0.437** | 0.377 |  | **-0.466** | 0.438 |
| Tea towel: 1st Change from neutral | -0.109 | **0.778** |  | 0.065 | **0.728** |
| Tea towel: 2nd Turns | -0.032 | **0.764** |  | -0.237 | **0.517** |
| Tea towel: 2nd Change from neutral* |  |  |  | 0.168 | **0.810** |
| *2nd change from neutral could only be used at 8M due to too few dogs (<5) exhibiting the response at 5M | | | | | |
