# Supplementary material for: An evidence-based decision assistance model for predicting training outcome in juvenile guide dogs
Source: PLoS One. 2017 Jun 14;12(6):e0174261. doi: 10.1371/journal.pone.0174261 (PMC5470660; doi:10.1371/journal.pone.0174261)
Supplement: S9 Table — Coef., test coefficients; these are correlation coefficients (rho) for all continuous, component or mean data and standardised test statistics for all tests with binary data from Mann-Whitney U tests (shown in italics). Significant associations are highlighted in bold. Associations in the direction opposite that predicted are shown in bold italics. * p<0.05, **p<0.01, ***p<0.001. (DOCX) [file pone.0174261.s009.docx]

**Supplementary Table 9.** Rotated component matrix loadings for the responses to subtests 8 (food), 9 (robin), 10 (pigeons) and 11 (human) from the juvenile guide dog behaviour test, at 5 and 8 months of age. The PCA’s achieved KMO statistics of 0.60 and 0.65 for the 5 and 8-month tests, respectively, with Bartlett’s test of spherictiy significant to p<0.001 for both. Cumulative variance explained by the components was 75.0% at 5 months and 63.2% and 8 months.

|  | **5M components** | | | |  | **8M components** | | |
| --- | --- | --- | --- | --- | --- | --- | --- | --- |
| **Variable** | **Food response** | **Human response** | **Pigeons approach 5M** | **Bird distraction 5M** |  | **Bird distraction 8M** | **Food response** | **Human response** |
| Food: Time oriented | **0.876** | 0.105 | -0.136 | 0.051 |  | 0.022 | **0.839** | -0.065 |
| Food: Pull strength | **0.875** | -0.076 | 0.228 | 0.022 |  | 0.232 | **0.804** | 0.174 |
| Human: Jumps | -0.147 | **0.882** | 0.108 | 0.09 |  | -0.018 | 0.235 | **0.841** |
| Human: Pull strength | 0.192 | **0.877** | 0.02 | 0.022 |  | 0.043 | -0.129 | **0.859** |
| Pigeons: Approach | -0.038 | -0.033 | **0.894** | 0.001 |  | **0.717** | -0.112 | -0.044 |
| Pigeons: Pull strength | 0.096 | 0.198 | **0.779** | 0.349 |  | **0.796** | -0.021 | 0.131 |
| Robin: Time oriented | 0.116 | -0.122 | 0.033 | **0.841** |  | **0.563** | 0.243 | -0.016 |
| Pigeons: Time oriented | -0.134 | 0.218 | 0.158 | **0.718** |  | **0.658** | 0.199 | -0.111 |
| Robin: Pull strength | 0.302 | 0.135 | 0.44 | **0.52** |  | **0.686** | 0.367 | 0.29 |
